# Flexible UWB and MIMO Antennas for Wireless Body Area Network: A Review

**DOI:** 10.3390/s22239549

**Published:** 2022-12-06

**Authors:** Vikash Kumar Jhunjhunwala, Tanweer Ali, Pramod Kumar, Praveen Kumar, Pradeep Kumar, Sakshi Shrivastava, Arnav Abhijit Bhagwat

**Affiliations:** 1 Department of Electrical and Electronics Engineering, Manipal Institute of Technology, Manipal Academy of Higher Education, Manipal 576104, India; 2 Department of Electronics and Communication Engineering, Manipal Institute of Technology, Manipal Academy of Higher Education, Manipal 576104, India; 3 Discipline of Electrical, Electronic and Computer Engineering, University of KwaZulu-Natal, Durban 4041, South Africa

**Keywords:** flexible antennas, implanted device, multiple input multiple output (MIMO), specific absorption rate (SAR)

## Abstract

In recent years, there has been a surge of interest in the field of wireless communication for designing a monitoring system to observe the activity of the human body remotely. With the use of wireless body area networks (WBAN), chronic health and physical activity may be tracked without interfering with routine lifestyle. This crucial real-time data transmission requires low power, high speed, and broader bandwidth communication. Ultrawideband (UWB) technology has been explored for short-range and high-speed applications to cater to these demands over the last decades. The antenna is a crucial component of the WBAN system, which lowers the overall system’s performance. The human body’s morphology necessitates a flexible antenna. In this article, we comprehensively survey the relevant flexible materials and their qualities utilized to develop the flexible antenna. Further, we retrospectively investigate the design issues and the strategies employed in designing the flexible UWB antenna, such as incorporating the modified ground layer, including the parasitic elements, coplanar waveguide, metamaterial loading, etc. To improve isolation and channel capacity in WBAN applications, the most recent decoupling structures proven in UWB MIMO technology are presented.

## 1. Introduction

Every year millions of people die from congestive heart failure, diabetes, paralysis agitans, cancer, emphysema, obesity, and many more chronic or fatal maladies [1]. The problem with all fatal infections is that many individuals manifest disorders only after it is too late to treat them. Wearable monitoring systems suitable for early diagnosis of anomalous diseases significantly enhance the quality of living and are a crucial solution for more cost-effective and preventive health care systems [2]. It can only be possible with a network of sophisticated, low-power sensing devices implanted/injected into the body or deployed on the body to provide timely information. These networks are usually known as wireless body area networks (WBAN) [1,2,3]. Body-centric wireless communication is considered a substantial subsystem for new-generation advanced consumer electronics. WBAN is likely to influence people’s approach toward fitness and health, akin to how the Internet affects their access/transfer of information. WBAN is formally defined by IEEE 802.15 as “a communication standard optimized for low power devices and operation on, in or around the human body (but not limited to humans)” [4].

**Figure 1:**
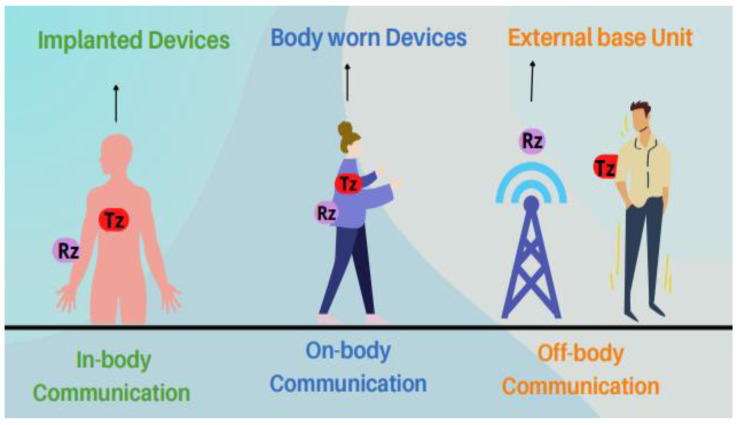
The classification of wireless body-centric communication.

Off-body, on-body, and in-body communication are the different forms of body-centric wireless communication. The classification of wireless body-centric communication is depicted in Figure 1, where Tz and Rz are transmitter and receiver antennas. The first type of communication occurs between an off-body device or system and an on-body network. The second type forms the wireless communication link between on-body networks and wearable devices. The third type is wireless communication between a medical implant and sensors network [5].

**Figure 2:**
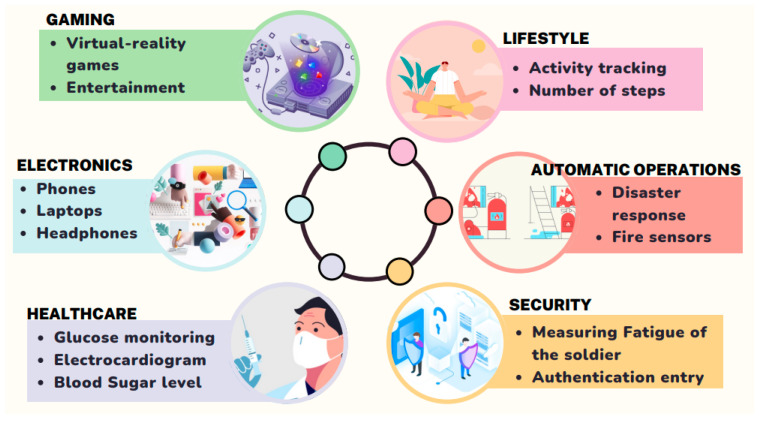
Applications of WBAN.

All WBAN applications focus primarily on enhancing the user’s quality of life and possess the potential to alter how individuals engage and benefit from information technology. There are numerous WBAN applications, which include medical and non-medical applications. A WBAN contains several portable, miniaturized, and autonomous sensors that can monitor human body activity, such as sporting, fitness, entertainment, health, and emergency applications. Some of the crucial applications of WBAN are illustrated in Figure 2.

**Figure 3:**
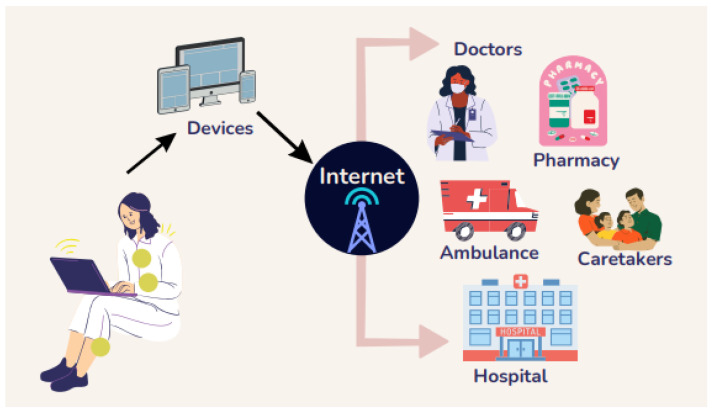
A real-time telemedicine infrastructure.

Miniaturized sensors and devices for monitoring, diagnostic, and therapy purposes, as well as wireless technological breakthroughs, have ushered in a new age in the battle to address healthcare challenges [1,2,3,4,5,6]. The antenna is crucial in body-centric wireless communication because it works as a transceiver antenna. If the antenna’s performance is poor, it will impact the system’s overall performance. Wireless devices deployed in and around the body are also crucial technology offering unparalleled portability for monitoring patients’ fettle in the hospital while traveling and at home. Figure 3 depicts a telemedicine system for real-time patient treatment. It demonstrates implantable or wearable wireless sensors that monitor, analyze data, and communicate a patient’s condition (such as pulse rate, pH level, body temperature, respiration, blood pressure, and oxygen saturation) without affecting the user’s daily activities. The collected information can be sent in real-time to an infirmary, health center, or primary repository via a local area network (LAN), wireless area network (WAN), or mobile network. Remote access to these data allows physicians to evaluate the patient’s health and alert through SMS or reminder messages [3,7].

The non-medical applications of WBAN are personal item tracking, real-time streaming, virtual reality, and gaming purposes (game control with hand gestures, mobile body motion games, and virtual world games) [1,7,8]. Innovative applications include smart meters, smart multimedia, home automation [9], and non-medical crises such as fire alarms and disaster response systems to enhance rescue and search operations [1,10]. WBANs provide better supervision of army activity on the battlefield, therefore assessing troop weakness and combat preparedness. A WBAN device can also be used to track an athlete’s performance and aid them during their course of practice [1,11].

Among the many subsystems of the WBAN, the most fundamental subsystem is the antenna, which serves as a framework for information exchange. The key challenge in antenna design is the need for flexibility due to variations in antenna topology due to the shape of the human body. When such antennas are flexible, it provides greater comfort to the users who wear them. Flexible antennas are emerging as viable candidates for the next generation of smart electronics, offering numerous advantages over their rigid antenna counterparts. Flexible antennas are notable for their lightweight, easily conformal nature, portability, small size, and high level of flexibility and energy efficiency [6,12]. Flexible antennas should have good mechanical properties, making them suitable for wearable electronics and medical applications. These mechanical properties allow the antenna to be ideal for the body area network because continuous bending of these antennas should not degrade their performance. Furthermore, the distance between the antenna and the human body frequently varies with human motion [13]. Additionally, the design of WBAN antennas is challenging due to various requirements such as a reliable link, a compact size, robustness in close proximity to the body, and minimal interference with other equipment. Future WBAN applications require broad bandwidth (BW), multiband operation, and high gain to connect body-worn sensors and remote processing units [1,2]. In addition, all WBAN devices must be able to transmit 0.1 mW of power, and the maximum radiated transmission power must be less than 1 mW, so that it complies with the FCC’s SAR of 1.6 W/kg in 1 g of bodily tissue [1,2,3,4,5].

**Figure 4:**
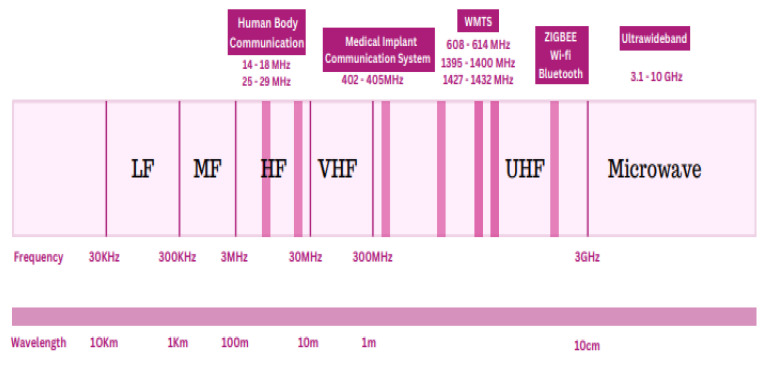
Wireless body area network bands.

The IEEE Standard 802.15.6 allocates the different frequency bands of the electromagnetic spectrum as depicted in Figure 4 for WBAN application [1]. Several bands can be used to design a flexible antenna, such as Medical Implant Communication Service (MICS) (402–405 MHz). It was originally designated for communications between implantable devices and devices on the body. The main benefit of this frequency range is its better transmission through human tissue. However, its main disadvantage is its 3-MHz bandwidth, which is inadequate for high-speed communication [14]. Wireless Medical Telemetry Service (WMTS) (608–614, 1395–1400, 1427–1432 MHz) is a licensed band used for the medical telemetry system. However, only authorized users, including physicians and qualified technicians, can utilize this band. Furthermore, the restricted WMTS (14 MHz) bandwidth cannot support video and voice transmissions [15]. The unlicensed Industrial, Scientific, and Medical (ISM) (433.1–434.8, 868–868.6, 902.8–928, 2400–2500, 5725–5850 MHz) is used for communications between on-body or off-body devices and in-body devices. Nevertheless, this band is currently overcrowded due to wireless personal area network and wireless local area network communications. Hence, other wireless systems may interfere with these devices. Additionally, this frequency spectrum cannot achieve the data rates offered by present digital networks [15,16]. For WBAN devices, there is still an increasing demand for high data rates to provide superior service quality.

To cater to these demands over the last decade, the ultrawideband (UWB) frequency band has been explored for short-distance and high-speed communications. UWB signals operate between 3.1 and 10.6 GHz with a minimum BW of 500 MHz [16]. Nonetheless, its numerous benefits, such as antenna miniaturization, high-speed communication, and low power consumption, have made it a better candidate for WBAN applications [17]. Furthermore, UWB transmitters do not cause significant interference to other radio devices. Furthermore, they do not pose any hazard to human safety. Impulse radio (IR) UWB transmitters and receivers have a simple design and are also power efficient [18]. Conversely, these systems have some demerits, such as multipath fading due to diffraction and reflection of the signal, scatter in angle of arrival, and long synchronization time. To address the issue of multipath fading, multiple input and multiple output (MIMO) can be integrated into the UWB system [19].

In this paper, a comprehensive exploration of the flexible substrates used in the design of antennas for WBAN applications is proposed. The main contribution of this article is threefold.

1. Firstly, a detailed study of available flexible substrates in literature is carried out, which forms the basis of designing the flexible antennas for WBAN applications. In addition to this, detailed advantages and disadvantages of these substrates based on their operational performance are presented.
2. Secondly, various flexible UWB antenna design techniques are reviewed, and their operational performances are discussed.
3. Thirdly, to enhance the data rate and improve isolation between the components, flexible UWB MIMO antenna design techniques are reviewed for various antennas available in the literature. Their summarized results are analyzed and presented. This review paper will help WBAN antenna engineers to design their antennas accordingly

This paper is organized as follows. We provide an exhaustive review of flexible materials used in various antenna designs for wireless communication. Second, several types of flexible UWB antennas based on the flexible substrates are used for in-body, on-body, and off-body wireless communication are explored. Thirdly, various flexible UWB MIMO antennas for WBAN will be discussed and compared. Flexible antennas are manufactured using substrates that are easily implemented into various irregular surfaces while retaining their functional characteristics. The following sections will concentrate on the most commonly used materials and their roles in the substrate and conductive material.

## 2. Flexible Material

**Figure 5:**
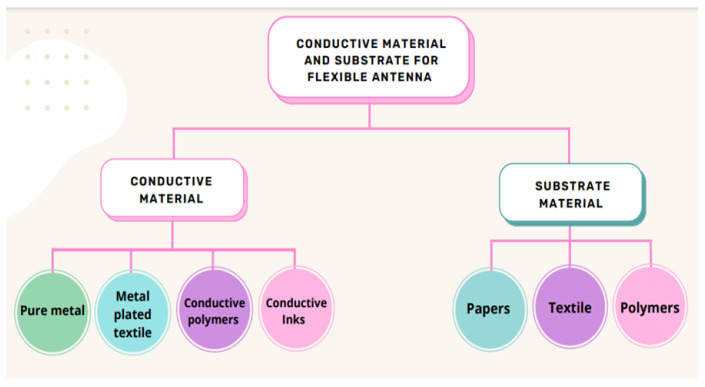
Materials for flexible antenna.

Various conductive materials and substrates are used to fabricate flexible antennas. The substrate is chosen based on its dielectric properties, miniaturization, mechanical pliability, susceptibility, and external environment endurance [12,13]. In addition, antenna performance, such as radiation characteristics, is decided by the choice of conductive material according to its conductivity. Materials suitable for use as a conductive layer, in general, include pure metals, metal-plated textiles, conductive polymers, and conductive inks [20]. A low-loss substrate is required for the design of flexible antennas. The substrate is chosen in such a way that it improves antenna efficiency when placed on the body. Polymers, paper, foam, and textile materials, are the most commonly used substrate in fabricating flexible antennas. Since flexible electronics are becoming more popular in WBAN, the substrate should conform to physical deformation [21]. Figure 5 shows the various conductive materials and substrates used to fabricate conformal antennas.

### 2.1. Conductive Materials

High electrical conductivity and low resistivity materials are desired in wireless applications to achieve large gain, efficiency, and bandwidth. [12]. Another desirable property of the conductive material is its resistance to mechanical deformation, such as bending and crumpling, and is easily integrated with numerous substrate materials. Furthermore, it should be resistant to material degradation caused by external effects such as corrosion and oxidation. The element used for the conductor can be characterized as (a) pure metal, (b) metal-plated textile, (c) conductive ink, and (d) conductive polymers [12,21]. Table 1 depicts the conductivity of various conductive materials that are used in the design of a conformal antenna. Pure metals, such as copper (Cu), silver (Ag), and aluminum tapes [22,23] and copper sheets [24], have been widely used to fabricate flexible antennas. These conducting materials have good conductivity, are easily available, and have good radiation characteristics. Metal-plated textile, also known as “electro-textile” and “E-textile,” is another common conductor used for designing wearable antennas. Electro-textiles or conductive fabrics such as nylon coated with nickel (Ni)/copper/silver [25,26], nickel-copper coated fibers [26], meshed polyester fibers coated with nickel zinc-blackened copper [27], copper polyester taffeta [28], Zelt (tin/copper coated plain weave) [29], ShieldIt [6,30], Nora (three metalized layers Ni/Cu/Ag) [31], Flectron (Ni/Cu polyester metalized material) [32], and nonwoven conductive fabrics (NWCFs) [33] are frequently used to fabricate conformal antennas. These conductive materials are considered excellent choices to be integrated into clothes and body-worn electronics as they are also washable and reliable. However, the electro-textile antenna has poor radiation characteristics due to its lower conductivity [21].

Conductive polymers, such as polyaniline (PANI) [34], polypyrrole (PPy) [35,36], and poly(3,4- ethylene dioxythiophene) polystyrene sulfonate (PEDOT: PSS) [36], are viable candidates for integration into antenna designs due to their low price and high flexibility. A drawback of the conductive polymer is its relatively low conductivity, which can be enhanced by adding single wall carbon nano tube (SWCNT), multiwall carbon nano tube (MWCNT) [37], and platinum-decorated carbon nanoparticle embedded polyaniline (Pt_C/PANI: CSA) [38]. Carbon or metal particle-based conductive ink is a potential material for the wearable antenna. Silver nanoparticle inks have also received attention due to their superior conductivity and chemical stability [12]. However, due to the high price of silver, its use is significantly restricted. Other inexpensive conductive inks are composed of copper and aluminum nanoparticles. However, copper nanoparticle inks oxidize quickly in ambient conditions and at temperature > 150 °C, which is required for inks to sinter successfully [39]. Graphene-based ink is an alternative to metallic conductive inks for its relatively excellent conductivity, environmental tolerance, and better system integration that requires flexibility. It also improves the device’s durability and prevents high-level deformation discontinuities [40,41]. However, inkjet printing for radiofrequency applications is complex as it requires precise control to achieve the required conductivity and surface roughness [12].

### 2.2. Substrate Materials

**Figure 6:**
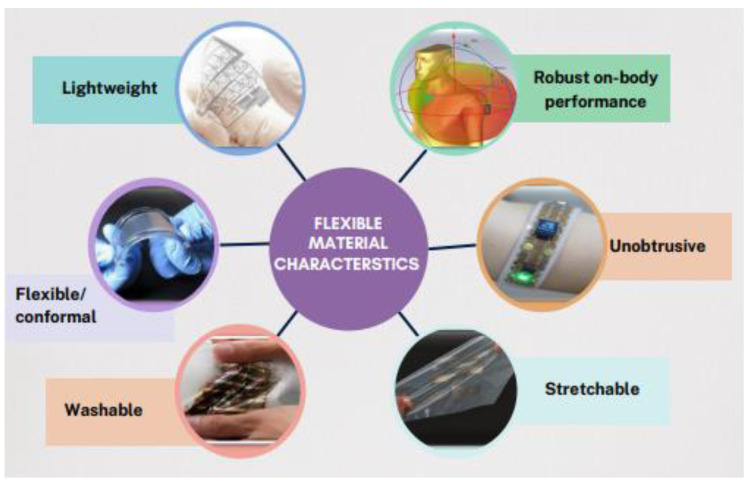
Flexible material characteristics.

Substrate materials are primarily used to support the antenna’s patch and ground plane. A flexible antenna design requires a substrate having low dielectric loss tangent (tanδ) and relative permittivity (*ε_r_*) up to several GHz [6]. The flexible substrate should also have lightweight, superior mechanical properties, be insensitive to temperature, unobtrusive, and stretchable, as shown in Figure 6 [13,24,29]. Another desirable property of dielectric materials is that they are not affected by material degradation or moisture absorption.

The dielectric material used for the design of flexible antennas can be classified as (a) paper, (b) textile, and (c) polymers. Other flexible substrates are also used in the flexible antenna design. Paper is an eco-friendly, renewable organic, and one of the most appealing materials for microwave applications. Paper is also incredibly cheap due to its widespread use in various industrial applications. In addition, compared to other biodegradable substrates, such as polylactic acid (PLA), the paper does not deform when heated, which is beneficial when curing printed conductive ink tracks [42]. Kodak photo is used to design conformal UWB antenna fed by coplanar waveguide (CPW) for Internet of Things (IoT) applications [43]. Xiaotian Li et al. [42] demonstrated another screen-printed radio frequency identification device (RFID) reader antenna system using HP’s Q8698A photo paper. However, due to defects formed in the coating applied to the surface of the photo paper, the printed antenna cannot be completely flexible. To overcome this problem, the synthetic paper material is used in the design of the antenna [44]. Another disadvantage of the paper-based antenna is that its relative permittivity can change depending on the surrounding environment, such as temperature and moisture [42,43].

Clothing material (nonconductive fabric) can be utilized as an antenna substrate, particularly when paired with textile conductors plated with metal. The textile antenna also provides more compatibility than the other wearable antenna as these materials are easily accessible and widely used in daily life by all humans and so they are regarded as a viable resource for the design of the flexible antenna. Textile antennas have been developed using numerous fabrics such as denim [6,23], plain woven polyester fabric [28], felt [29], acrylic fabric [31], cordura fabric [45], and fleece for various applications. However, due to the continuous exchange of water with their surroundings, textile materials’ relative permittivity and loss tangent are subject to significant change. Moreover, textiles are porous and anisotropic materials with varying densities and thicknesses depending on fabrication (crochet or woven), component materials, and consistency. These processes, which significantly impact the dielectric characteristics of fabrics, are challenging to manage in practical applications [46]. Table 2 depicts the dielectric constant, dielectric loss, and thickness of commonly employed substrates for fabricating flexible antennas.

In recent years, the elastomer or thermoplastics substrate has also gained popularity because of its toughness, flexibility, wettability, and stretchability. Polymer-based materials, such as liquid crystal polymers (LCP), polydimethylsiloxane (PDMS), polyethylene (PE), polyethylene terephthalate (PET), polyimide (PI), and poly(tertiary fluoroethylene) (PTFE), proved to be excellent candidates for the development of conformal antennas. [13,24]. In [47], the authors describe a flexible UWB coplanar waveguide-fed antenna fabricated using LCP. It is an organic, lightweight material that can form multilayer configurations at reasonably low temperatures, enabling active devices and circuits to be embedded [47,48]. Polydimethylsiloxane is becoming increasingly popular as a substrate due to its superior conformability, lower cost, and better binding strength. Additionally, it can withstand extreme environments, has chemical stability, good electrical insulation, and biocompatibility [49]. In addition, it is water-repellent, translucent, and resistant to temperatures up to 200 °C [24,25,26]. In the paper [26], a flexible UWB antenna is discussed, with manufacturing centered on the PDMS conductive fabric composite method. In [24], the authors developed a conformal UWB antenna based on PDMS material for WBAN application. Materials with variable relative permittivity are required for smart electronics applications. Therefore, various fillers are mixed with PDMS to enhance their properties. By using metal and ceramic reinforcements with various relative permittivity values, dielectric values of PDMS can be manipulated across a wide range [49]. PDMS, microscale aluminum oxide (Al_2_O_3_), and graphite (G) granules are used as fillers with variable weightage to vary and regulate the substrate’s characteristics. It achieves a better match with the human and reduces the antenna size while retaining the PDMS’s adaptability [49,50]. The authors modified the PDMS substrate by mixing it with glass microspheres (GM) to lower its dielectric permittivity and achieve good radiation characteristics [49,51].

PTFE is widely utilized as a substrate material in electronic industries due to its superior electrical and mechanical qualities, ultimate tensile strength, biocompatibility, high and low temperature, corrosion resistance, and stable dielectric constant across a broad frequency range [52]. The author designed a CPW-fed fishtail-shaped antenna for dual-band applications using a PTFE substrate [52]. However, the processing techniques for PTFE polymers, such as melt extrusion and injection molding, are complex [49,53]. Various impregnation and sintering techniques are used to improve the mechanical and radiation characteristics of a 3D-integrated composite antenna made of glass fiber and PTFE resin [53]. In [54], the authors describe nanofiber composite membranes with improved conductive, mechanical, dielectric, and crystalline properties. Due to their better electrical, mechanical, and moisture resistance, PET and PEN are used in numerous conformal antenna designs [13,49]. A previous article [55] described a flexible and biocompatible ultrahigh-frequency meander antenna operating at about 800 MHz on a PE substrate for surface acoustic wave sensors. Compared to textiles, PET substrate-based antennas are less impacted by wrinkles, moisture absorptions, and pattern flaws [56,57]. Kapton polyimide (KP) is also utilized in designing flexible antennas due to its outstanding mechanical, chemical, and electrical properties over a broad frequency range [34]. In addition, Kapton is available in low thicknesses and has high tensile strength, dielectric strength, and thermal rating. In [12], the authors designed a flexible UWB fed by a linear CPW based on Kapton polyimide.

The study indicates that a flexible patch antenna requires high-conductive and low-loss dielectric materials for efficient electromagnetic radiation transmission. Additionally, the flexible substrate should be elastic, have excellent mechanical properties, be temperature-insensitive, and be lightweight. Popular and desirable flexible substrates include PDMS, paper, PET, Kapton polyimide, PEN, and liquid crystal polymer.

**Table 2:** Comparison of various substrate materials in flexible antenna design.

Substrate	Dielectric Constant $\left(\varepsilon_{r}\right)$	Dielectric Loss (tanδ)	Thickness (mm)	Ref.
**(a) Paper**				
Photo paper	3.2	0.05	0.44	[42]
Kodak photo	2.85	0.05	0.254	[43]
Synthetic paper	2	0.0022	0.26	[44]
**(b) Textile**				
Denim	1.67	0.0035	2	[6]
Plain woven polyester	2.193	0.004	0.5	[28]
Felt	1.22	0.016	2	[29]
Acrylic fabric	-	-	0.5	[31]
Cordura fabric	2.05	0.025	2	[45]
**(c) Polymers**				
Liquid crystal polymers	3	0.002	0.225	[48]
Polydimethylsiloxane (PDMS)	2.7	0.134	1.5	[24]
Polyethylene (PE)	2.82	0.005	0.125	[55]
Polyethylene terephthalate (PET)	3.16	0.0071	0.125	[56]
Polyimide (PI)	2.91	0.005	0.2	[13]
Poly(tetrafluoroethylene) (PTFE)	2.2	0.0009	0.127	[52]
Kapton polyimide (KP)	3.4	0.002	0.0508	[12]
PDMS-Al_2_O_3_-G	15.8	0.052	3.57	[50]
PDMS-GM	1.85	0.014	3	[51]
PTFE/E-glass	2.5	0.003	4	[53]
PTFE/CNT	-	-	2	[54]

## 3. Flexible UWB Antenna Design Strategies

In contrast to the narrowband counterpart, the design of UWB antennas depends on both radiation characteristics and their ability to maintain signal shape as it uses unconventional carrier-free modulation in impulse radio systems [17,18]. Since ultrawideband systems demand very different antenna topologies and propagation properties than narrowband systems, practical antenna design is more complicated. The antennas must work efficiently throughout the BW and be unaffected by the user’s morphology or the antenna’s location on the body [6]. Therefore, it is essential to ensure that the antenna’s far-field radiation characteristics intended for on-body application are relatively the same on bending [24]. Additionally, wearable designs must adhere to SAR requirements. The antenna must be robust and less susceptible to deformation when its dimensions are small. Miniaturization has therefore been crucial in enhancing the performance of wearable antennas [58]. Due to their straightforward design, large bandwidth, and effective radiation, planar monopole antennas are often used in the design of UWB communication systems. In Table 3, a detailed comparative study of the conformal UWB antennas, based on their performance characteristics in terms of bandwidth, dimension, SAR, peak gain, and merits and demerits, is included and presented [6,24,26,28,29]. In this section, several flexible UWB antenna designs are illustrated.

A wearable low-profile UWB organic antenna fed by a coplanar waveguide is proposed in [34]. The antenna is developed using PANI/MWCNTs with a conductivity of 4500 S/m and the patch is elliptical in shape. The substrate is 130 μm thick KP having dielectric properties as *ε_r_* = 3.48 and tanδ = 0.002. After the simulation, the antenna is placed on a cloth to examine the impact of bending along the different axes and crumpling. The uncrumpled antenna has a good reflection coefficient (S11) and a BW of 1–8 GHz but there is a shift in the resonant frequency to the lower band and an impedance mismatch. The peak gain measured at 5.8 GHz is 1.86 and 3.1 dBi for uncrumpled and crumpled antennas. The far-field radiation at the resonating frequencies are omnidirectional (OMD) and bidirectional (BD) patterns in the E and H planes for both crumpled and uncrumpled antenna. The cross-polarization patterns for the crumpled antenna showed a dipole characteristic, with the minimum gain in the E-plane at 0° and 180°, and the peak gain at ±90°. Therefore, the antenna demonstrated promising outcomes for wireless communication, particularly when included in clothing. However, the SAR analysis is not evaluated.

**Figure 7:**
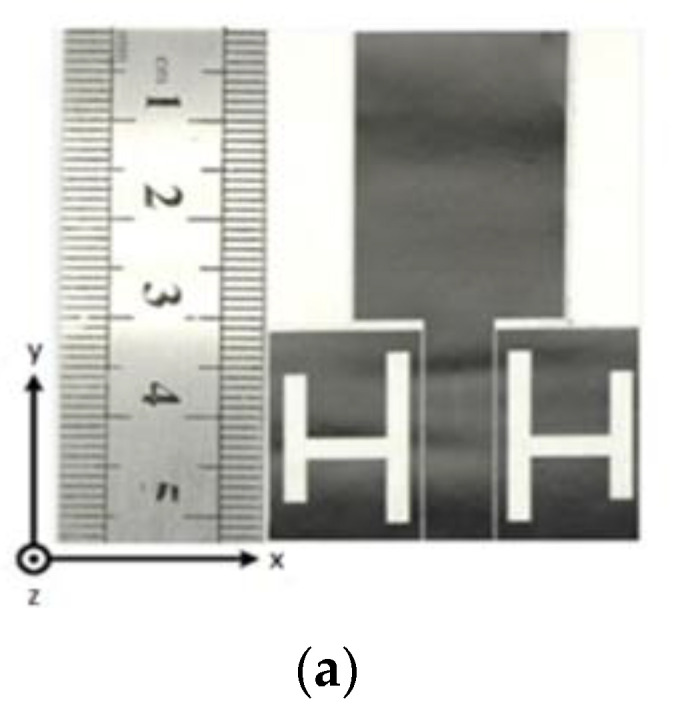

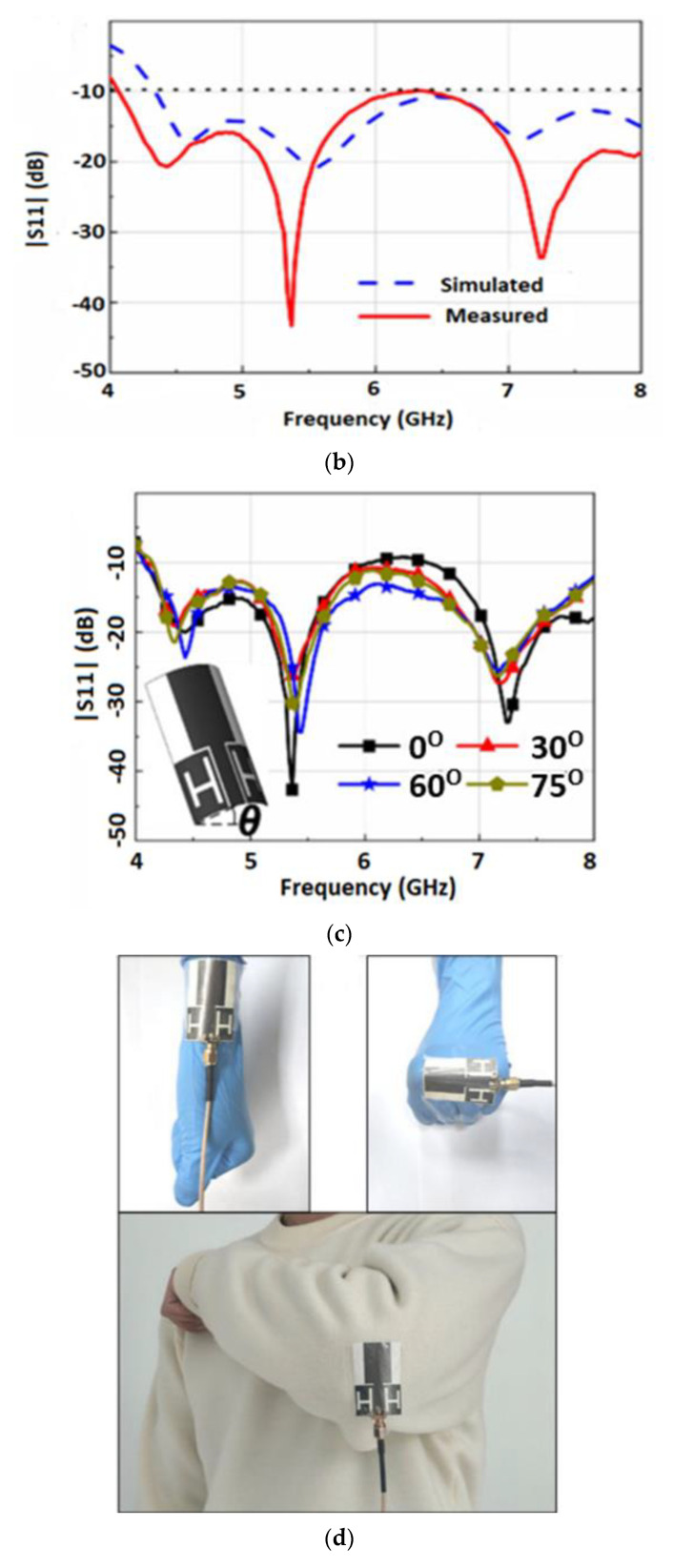

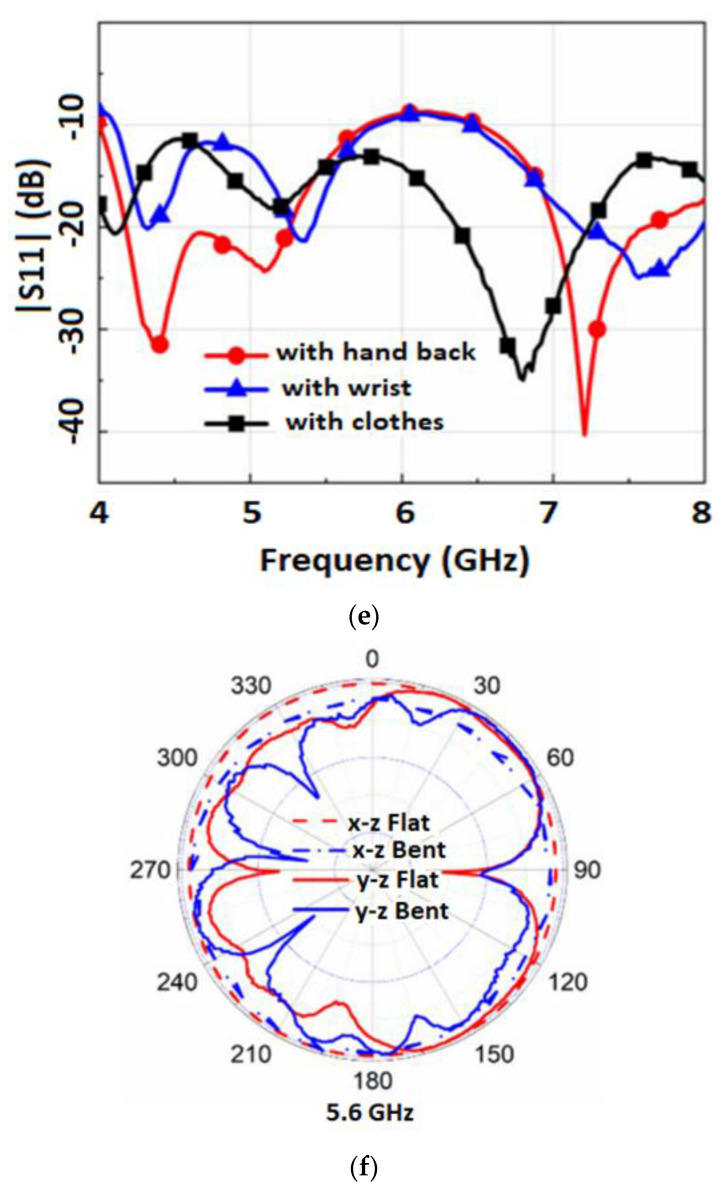
(**a**) The GAF antenna prototype. (**b**) |S11| of the antenna. (**c**) |S11| of the antenna under bending. (**d**) Antennas under different bending scenarios. (**e**) |S11| curves when attached to the body. (**f**) Radiation pattern. Reprinted (**a**–**e**) from Ref. [59].

In [59], the author presented a CPW-fed conformal wearable antenna fabricated using super-flexible composite ceramic material having a thickness of 255 mm, *ε_r_* = 3.2, and a high conductive graphene-assembled film (GAF) with a resistivity of 10^6^ S/m. The suggested antenna has a rectangular patch. Two H-shaped slots are cut into a CPW structure to alter the surface electric current density and increase the BW of the antenna, as illustrated in Figure 7a. The antenna with a relatively compact size has an impedance BW from 4.3 to 8.0 GHz, as seen in Figure 7b. The characteristics of UWB are analyzed, and conformality experiments at various bending angles are conducted. The antenna has a BW of (4.1–8.0 GHz) when bent, with a peak gain of 3.9 dBi in its flat state and 4.1 dBi in its bent form, as depicted in Figure 7c. The resonance frequency of the antenna shifts substantially once the antenna is mounted to the wrist, hand, or clothing. However, the |S11| values are below −10 dB (Figure 7d,e). The antenna’s far-field radiation characteristics for bent and flat configurations are also measured. Figure 7f illustrates how the beam becomes more focused toward the desired directions when the antenna is bent, resulting in a narrower back lobe. The GAF antenna’s resonance frequency differs slightly from the simulation’s output and is unable to cover the entire UWB spectrum.

Wang, Z. et al. [60] developed a compact elliptical-shaped patch fed by CPW techniques. The ladder-shaped ground plane is printed on a 50 μm thick polyimide substrate. Silver with a thickness of 7.54 μm and average resistivity of 4.92 × 10^−5^ Ohm-cm is used as the conductive material. The substrate material has a tanδ of 0.001 and *ε_r_* of 3.5. The ground planes and feed line structure are optimized for UWB and impedance matching. The proposed antenna’s simulated and measured BW are 1.40–16.40 GHz and 1.35–16.40 GHz, respectively. These frequencies cover the entire standard UWB spectrum. The realized gain exceeds 2.8 dB in the UWB band region and a maximum of 5.19 dB at 5.5 GHz. The antenna’s far-field radiation patterns at 2.45 and 5.2 GHz are omnidirectional. The antenna conformability is tested by bending it using foam cylinders. Under various bending situations, the antenna’s radiation pattern has significant ripple. The antenna shows a maximum radiation efficiency of 86% throughout the BW and a minimum value of 60%. The antenna has minimal bending susceptibility; however, the SAR analysis is not studied.

Another UWB-flexible antenna with a permittivity of 3.5 and a thickness of 70 μm is designed and developed [61]. The antenna has a hybrid-shaped patch fed by the CPW technique. Additionally, to enhance the BW, the feeding line has a different height and is etched with an arc shape on both sides of the upper-right corner of the left ground. The flat antenna displayed a 3.06–13.58 GHz impedance bandwidth and more resonance at 15.9–20.5 and 20.9–20.2 GHz. In the frequency range of 3 to 18 GHz, the antenna obtained a maximum gain of 1.69 dBi and an efficiency greater than 59%. The antenna is bent on cylindrical foam to test the conformability. The bent antenna’s bandwidth ranges from 2.8 to 13.55 and 16.6 to 22 GHz for a radius of 20 mm, and from 3.1 to 12.8 to 16.7 to 22 GHz for a radius of 10 mm. However, bending affects the radiation pattern, which becomes more assertive with a smaller bending radius. The far-field radiation pattern of the antenna is omnidirectional in situations of flat and bent states.

In [26], a UWB monopole with a circular path is presented for wearable applications having a BW of 2.85 to 8.6. The antenna is fabricated using the PDMS conductive fabric composite technique. The radiator is composed of a nickel-copper- silver-coated nylon ripstop with a thickness of 0.13 mm, and the ground plane is composed of nickel—a copper-coated ripstop with a thickness of 0.08 mm. PDMS has *ε_r_* of 2.77, increasing tanδ from 0.02 to 0.076 from 2 to 10 GHz. The patch consists of an annular-circular ring that is loaded with two rectangular cuts. To accomplish UWB, two other parasitic rings are concentrically inserted around the circular patch. The antenna’s −10 dB simulated and measured impedance bandwidths are 2.95–9.2 GHz and 2.85–8.6 GHz, respectively. The antenna bending experiments are performed at a 40 mm radius in both the x- and y-axis directions to confirm the antenna’s conformability. There is a shift in the resonant frequency and an impedance mismatch on bending. The antenna’s far field pattern is OMD in the x-y plane and BD in the x-z plane. The antenna gain varies between 2.9 and 6.2 dBi within the working bandwidth. For flat and bent circumstances employing Gaussian signals, the system fidelity factor is higher than 86%, which is adequate for accurate transmission.

**Figure 8:**
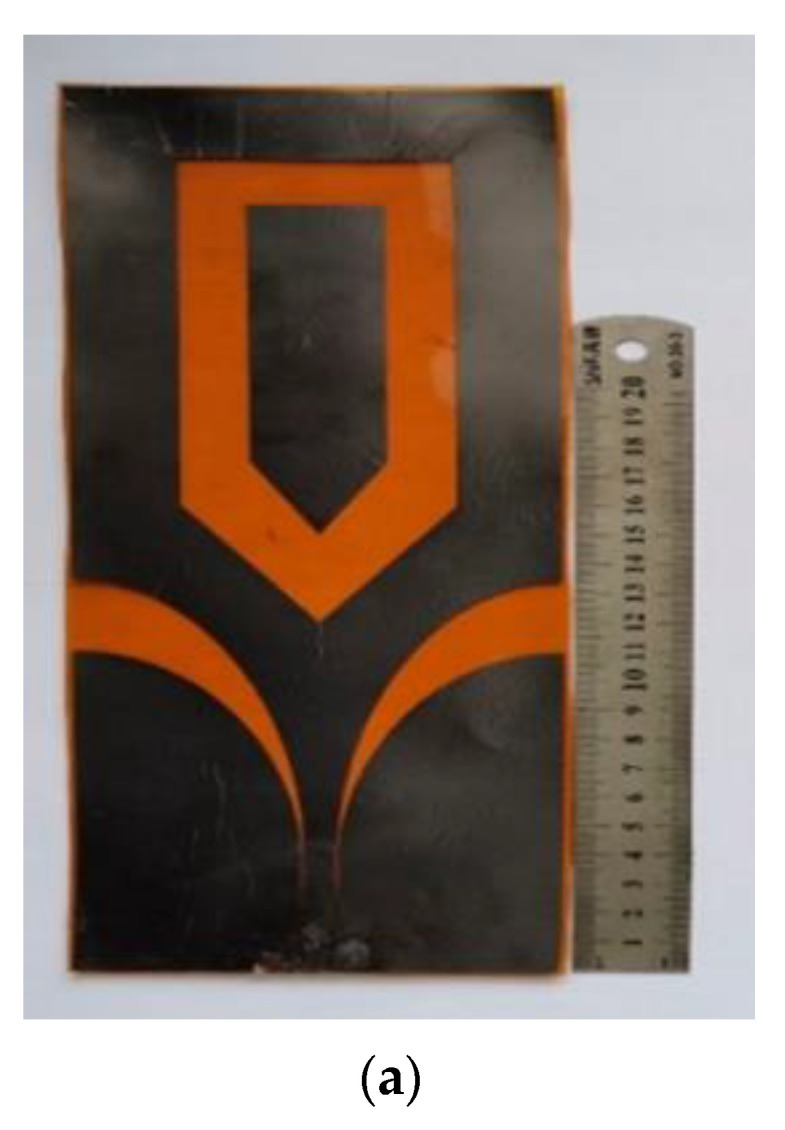

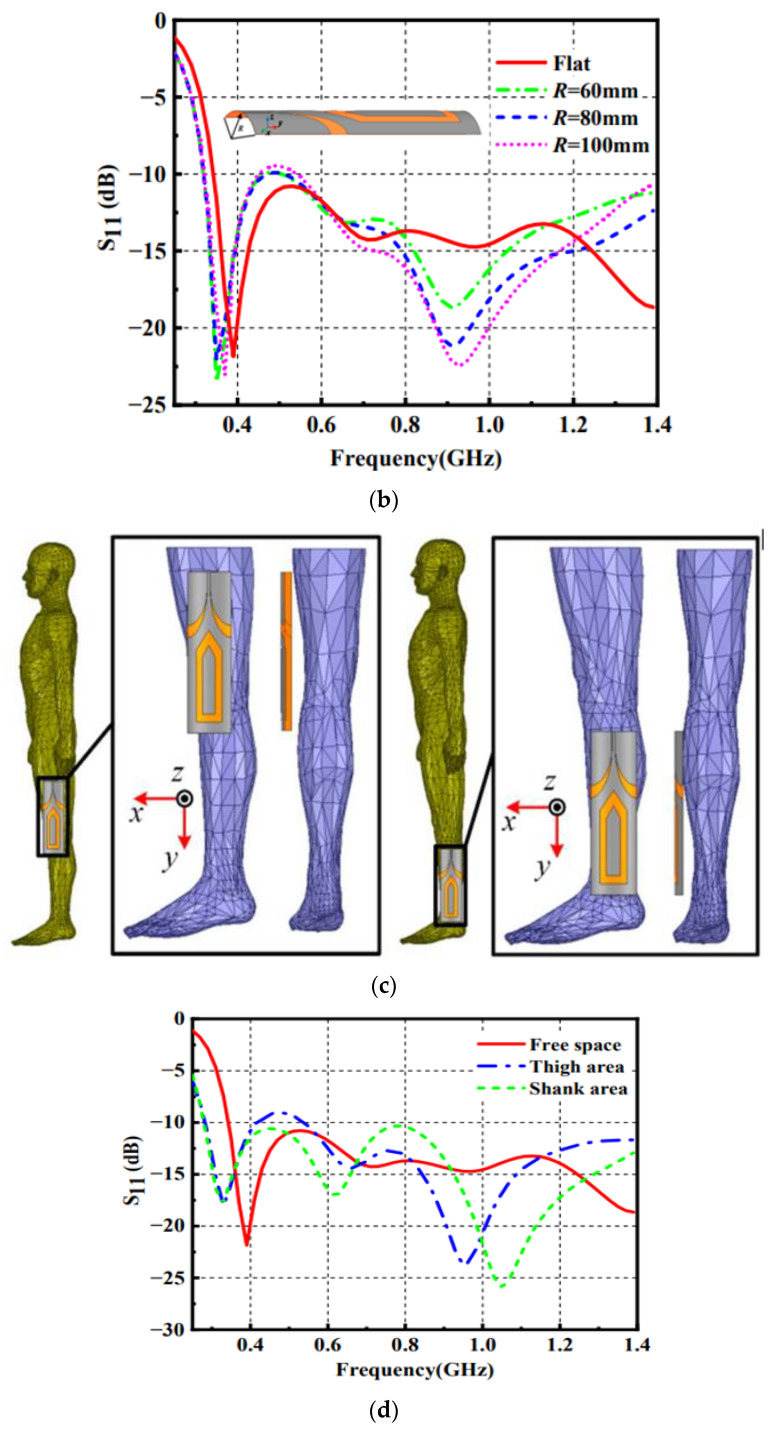

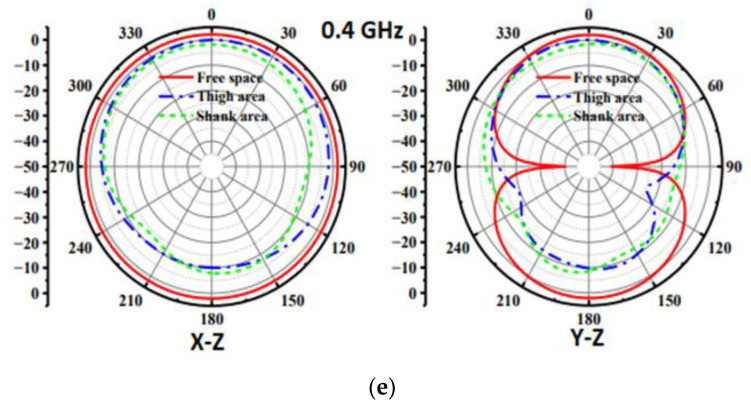
(**a**) Fabricated UWB antenna. (**b**) S11 of the antenna. (**c**) The antenna loaded on the different positions of the voxel model. (**d**) S11 of the loaded antenna. (**e**) Radiation pattern. Reprinted (**a**–**e**) from Ref. [62].

Li et al. [62] developed a flexible UWB antenna for wearable on-body devices for 0.34 to 1.4 GHz bands. As depicted in Figure 8a, polyimide with a width of 0.1 mm, *ε_r_* = 3.5, and tanδ = 0.0027 is selected as the substrate. Graphite films (dc resistivity σ = 1.1 × 10^6^ S/m), having a thickness of 26 μm, are used as flexible conductor material. The antenna is excited by a CPW for impedance matching. The UWB features of the antenna are achieved by incorporating a flaring ground with an arrow-shaped slot within the patch antenna. Slight degradation of the S11 around 0.5 GHz is observed when bending experiments are performed with different bending radii along the y direction, as illustrated in Figure 8b. As demonstrated in Figure 8c, a wearable antenna is loaded onto a voxel model’s thigh and shank region to examine the human body’s effect. S11 plots (Figure 8d) reveal a shift in the resonant frequency to the lower band and an impedance mismatch. The far-field radiation pattern of the antenna in the x-z plane is omnidirectional and an 8-shape pattern in the y-z plane when loaded near the human body, as presented in Figure 8e. However, the antenna efficiency reduces from 95% to 60% when operated around the human body, limiting the power transmission in free space.

The research presented an inkjet-printed UWB flexible antenna on photo paper (*ε_r_* = 2.85 and tanδ = 0.05) for wearable applications [43]. Silver nanoparticle ink is used for printing due to its high conductivity (25 mΩ^−^^1^/cm). A circular radiating patch with a double-stepped symmetric ground plane fed by a CPW technique is employed to enhance the BW. The impedance bandwidth of 3.2–30 GHz is noted for both simulations and measurements, with a gain of 4.87 dBi and an efficiency of 86.61 percent. The far-field patterns at the resonating frequencies are omnidirectional. When bent in horizontal and vertical directions, the S11 curves have limited variation.

Janapala et al. [24] designed a flexible UWB antenna for WBAN application in the 1.5–15 GHz band. The proposed antenna is fabricated using a PDMS substrate having *ε_r_* = 2.7, 1.5 mm thick, and a tanδ of 0.134. As the conducting layer, 0.193 mm thick copper foil is employed. A fork-shaped patch with a circle at the center, forming a crescent-shaped slot, and having a reduced ground plane is proposed to attain wide impedance bandwidth. The S11 is less than –10 dB when the antenna is placed on a human body, showing excellent on-body performance. However, some stopbands exist in some mid-frequency ranges. The antenna has a maximum gain of 6.76 dB in the frequency range of 1.5 to 15 GHz. The far-field patterns at various resonating frequencies with and without bending are OMD in the E plane. However, the radiation pattern changes to BD in the H plane. The obtained SAR is significantly below the FCC-mandated limit with values of 1.376 and 1.482 W/kg with and without bending at the resonant frequency, respectively. The resonant frequency of the antenna shifts under varying bending radii and nominal distances between the phantom model and the antenna on bending. The antenna’s physical dimensions of 67 mm by 44 mm do not fulfill the standards for the downsizing of a wearable antenna.

In [28], another UWB antenna for wearable microwave medical imaging is studied. Polyester textiles (*ε_r_* = 1.7 and tanδ = 0.004) with a width of 0.5 mm is utilized as a substrate material. Copper polyester taffeta fabric with a thickness of 0.08 mm (dc resistivity = 0.4 *×* 10^5^) makes up the antenna’s conducting layers. In the radiation patch’s lower corners and upper edge, two triangles and some parallel slots are cut to achieve UWB and reduced size. The S11 of the fabric antenna is comparable to the simulated S11, resulting in an ultrawide bandwidth of 109%. When bent on cylindrical foam along the y- and z-axes, the S11 curves exhibit negligible variation in resonance frequency. However, when the antenna is loaded on the phantom, the S11 measurement indicates that the antenna exhibits few stop bands. The far-field patterns at the resonating frequencies are OMD. Working near the human body, the antenna’s gain is 2.8 and 2.9 dBi at 1.2 and 2.4 GHz. The calculated SAR is far below the FCC-mandated limit, with values of 0.0014 W/kg occurring at 2 GHz on the surface of the phantom when the input power to the antenna is one milliwatt.

A transparent circular UWB dual-substrate-supported monopole antenna is reported in [63]. The UWB-flexible antenna is designed and fabricated on a soda lime glass substrate with a width of 2.2 mm, ϵ_r_ equal to 7.3, and tanδ equal to 0.04. Fluorine-doped tin oxide is used to develop the antenna’s conducting components (patches and ground), having a conductivity of 2.47 *×* 10^5^ S/m and thickness of 650 nm. The circular patch diameter, ground plane length, and feed line width are optimized to obtain a UWB bandwidth. Using dual substrates to increase the antenna’s bandwidth and the proximity coupling approach is employed to eliminate spurious radiation. The developed antenna operates at a frequency range of 4–8 GHz. The far-field pattern of the antenna is omnidirectional, with a peak gain of 1.2 dBi. However, the SAR and bending analysis are not evaluated.

In [64], a UWB screen-printed antenna is presented for a wearable application. Kapton (*ε_r_* = 3.5 and tanδ = 0.007), having 125 μm thickness, is used as a substrate material. The ink layer used has a conductivity of 1.7 × 10^7^ S/m and a thickness of 8 μm. The radiating structure comprises two inverted L-shaped elements, a matching stub, and DGS to obtain UWB. The suggested antenna has an impedance BW of 1.77–6.95 GHz and gains 2.5–5.9 dBi. Additionally, bending tests are conducted at various radii to ensure the conformability of the antenna. The antenna exhibits a relatively stable S11 under different bending situations. The unbent antenna’s far-field pattern is monopole-shaped in the E-plane and an almost omnidirectional pattern in the H-plane. However, when an antenna is bent, the radiation patterns change significantly.

**Figure 9:**
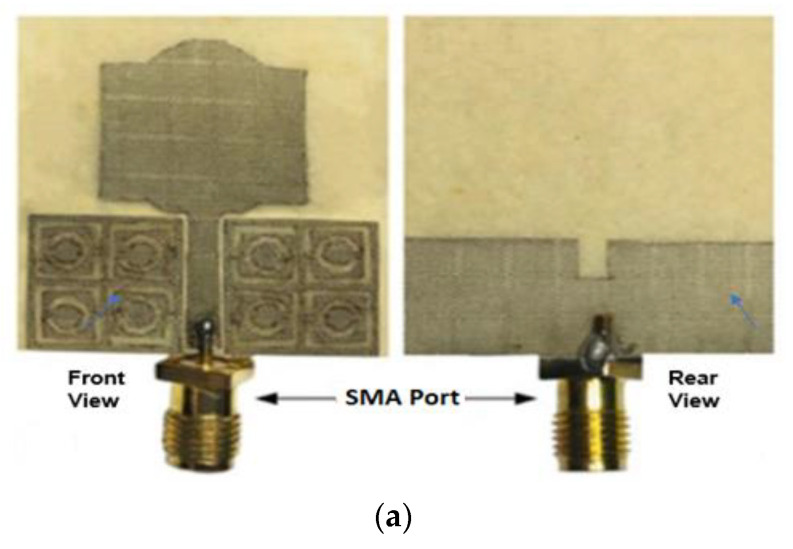

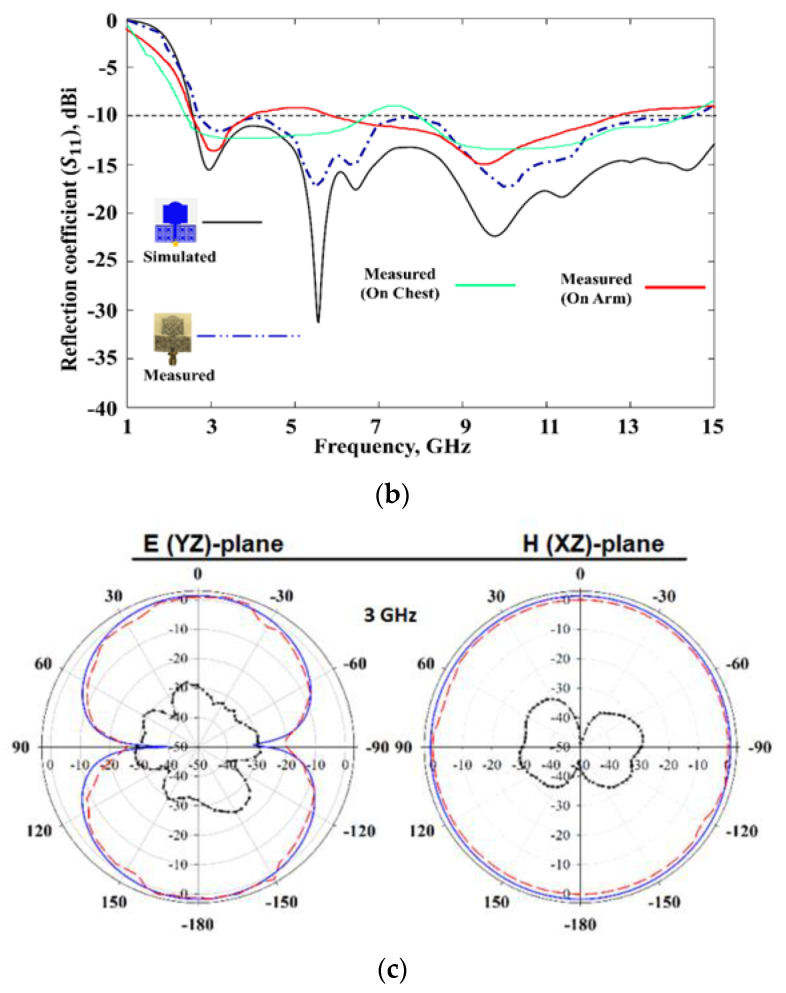
(**a**) Fabricated UWB antenna. (**b**) S11 of the antenna on-body and off-body. (**c**) Radiation patterns. Reprinted (**a**–**c**) from Ref. [65].

In [65], the literature indicates that metamaterial-based UWB antennas enhance antenna performance. The proposed antenna is fabricated using a viscose-wool felt substrate having *ε_r_* equal to 1.44, 3 mm height, and tanδ equal to 0.044. As a conducting layer, 0.17 m thick Shieldit SuperTM with a conductivity of 1.18 *×* 10^5^ S/m is used. As shown in Figure 9a, the antenna is designed using a combination of rectangular and half-elliptical patches. Additionally, two metamaterial unit cell arrays are positioned 0.4 mm on either side of the feedline to enhance the radiation characteristics. A DG plane is created on the opposite side of the feedline to achieve UWB. The prototype antenna is attached to the body to examine the impact on the human body. The finding indicated a −10 dB impedance BW from 2.55 to 15 GHz in calculations and from 2.63 to 14.57 GHz in measurements, as shown in Figure 9b. Simulations and experiments achieve peak gains of 4.84 and 4.4 dBi, respectively. At 3 GHz, an omnidirectional far-field pattern is seen. However, at higher resonating frequencies, an OMD pattern is observed in the H-plane, while a BD radiation pattern is seen in the E-plane, as seen in Figure 9c. There is no bending and SAR measurement analysis.

Defective ground planes or CPW techniques are usually favored because they permit easy BW enhancement. However, such designs are unsuitable for wearable devices for two primary reasons. Firstly, the DG plane implies that the high-permittivity and lossy biological tissues will severely load the antenna, compromising its performance. Second, these antenna configurations result in back radiation, which inevitably increases SAR within the human body. UWB antennas with full ground plane [66], and artificial magnetic conductors (AMCs) [29] are reported to increase gain. It also improves radiation characteristics and minimizes back radiation to mitigate the above problems.

Simorangkir et al. [66] presented a coaxial-fed planar UWB antenna designed for wearable applications in the 3.7–10.3 GHz band. The PDMS is used as a substrate layer having a *ε_r_* = 2.7 and a tanδ increasing from 0.02 to 0.07 over the BW of 3.68–10.6 GHz. Two arc-shaped patches are designed to obtain the upper and lower UWB frequencies. Further, two identical T-shaped slots are cut at the lowest UWB operating frequency to enhance matching. The antenna has a total efficiency of 27%, and a voltage standing wave ratio (VSWR) in the frequency range of 3.7–10.3 GHz less than 2 in both free space and a flat phantom. To examine the effect of bending, antenna prototypes are bent around the head and wrist of an anatomical phantom with varying radii along the axes. The VSWR performance of the bent antenna setups improved in comparison to the unbent antenna placed on the flat phantom. As a result of the full ground layer, the SAR value at different resonances are less than 2 W/kg. The antenna retains its OMD pattern in both free space and flat phantom. The total radiation efficiency of the antenna reduces when placed on the phantom. The antenna has a large dimension which does not fulfill the requirements for the miniaturization of wearable antennas. Since the antenna’s bending radius is relatively large, small radius bending should be considered.

**Figure 10:**
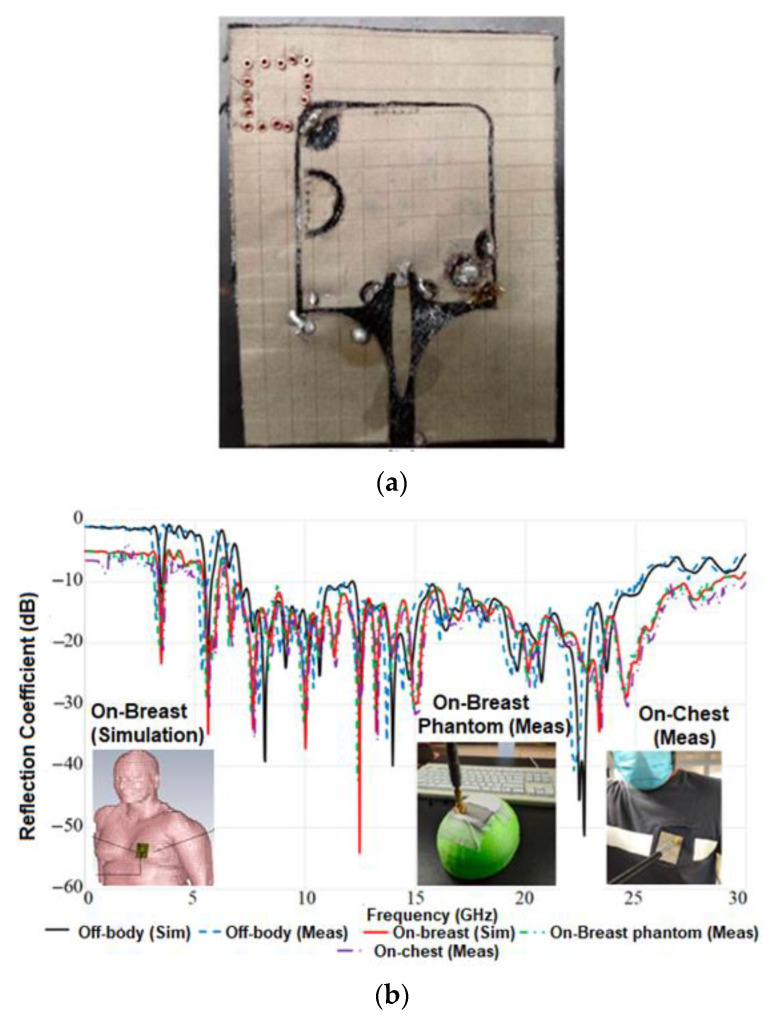

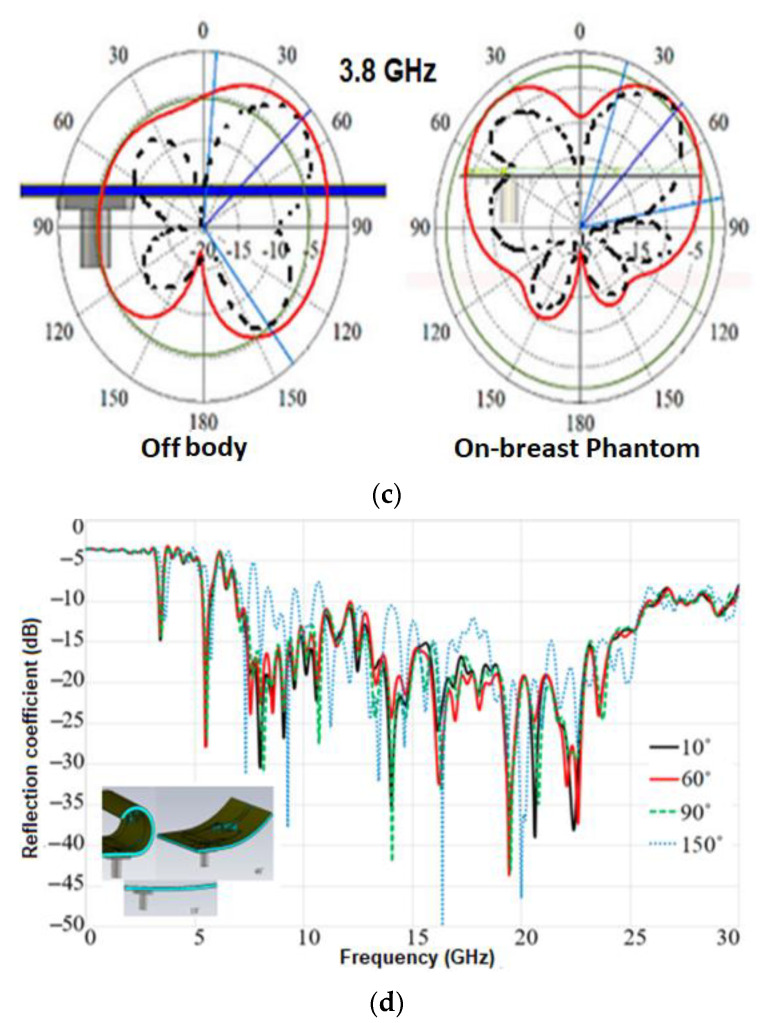
(**a**) Fabricated antenna prototype. (**b**) Radiation patterns. (**c**) S11 of the antenna on-body and off-body. (**d**) S11 results for different bending degrees. Reprinted (**a**–**d**) from Ref. [6].

A low-profile rectangular-shaped UWB with a complete ground plane is proposed for breast cancer and WBAN applications [6] as illustrated in Figure 10a. Denim (with *ε_r_* = 1.7 and a thickness of 0.7 mm) serves as the antenna’s substrate, whereas ShieldIt conducting (0.17 mm) conformal material serves as the conductor. The antenna is fed using a ground coplanar waveguide. The gain and bandwidth are improved using photonic band gap (PBG) structures and substrate-integrated waveguide (SIW) techniques. The antenna has a BW between 7 and 28 GHz, the maximum radiation efficiency of 96%, and a peak gain of 10.5 dBi. At the resonant frequencies. The far-field pattern of the antenna is OMD, as depicted in Figure 10b. The peak SAR findings due to the isolation offered by the ground plane, at 3.8, 5.8, 7, and 28 GHz, are found to be 0.25, 0.7, 1.29, and 2.04 W/kg, and 0.071, 0.171, 0.520, and 0.690 W/kg, below the permitted limit of 2W/kg. The resonance frequency of the antenna shifts substantially once the antenna is mounted on the body, as depicted in Figure 10c. The antenna is bent at an increasing angle to investigate the impact of antenna deformation. However, the |S11| value is not significantly influenced by bending, as seen in Figure 10d.

**Figure 11:**
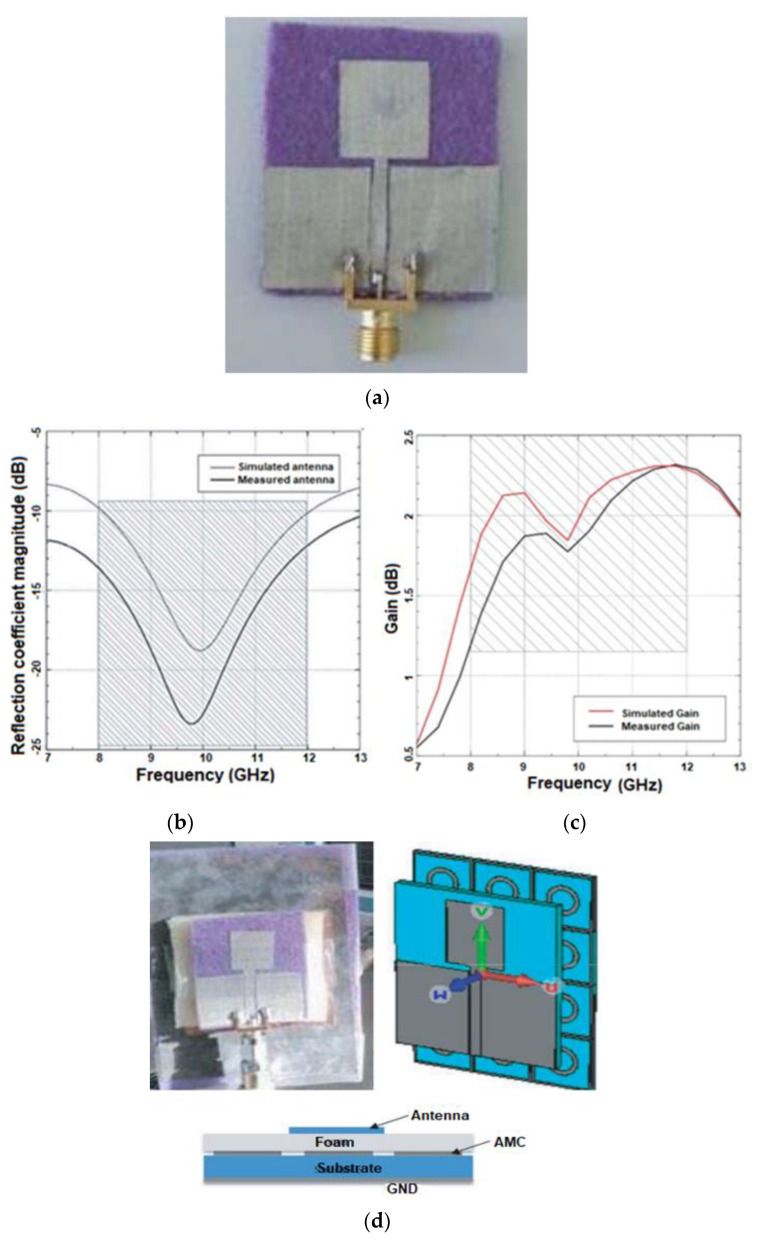

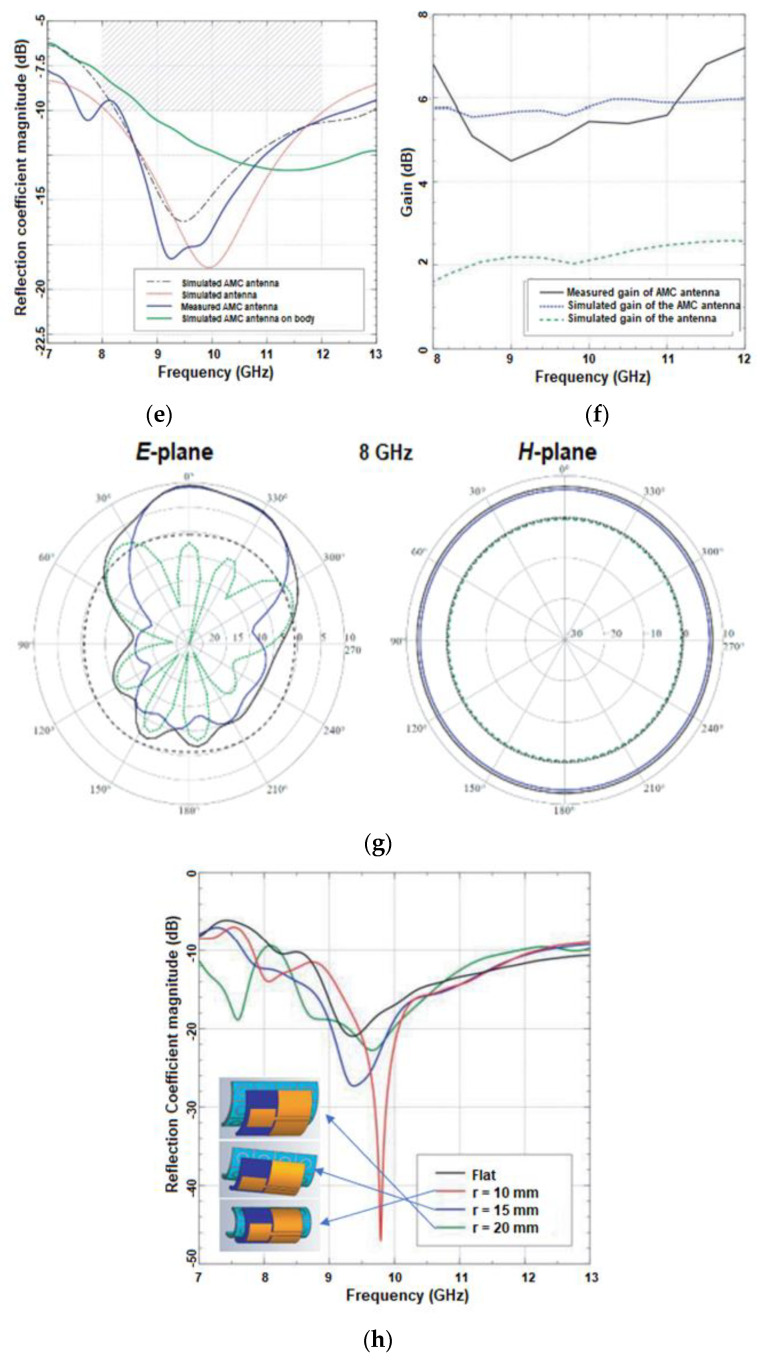
(**a**) Fabricated UWB antenna. (**b**) S11 of the monopole antenna. (**c**) Gain of the monopole antenna. (**d**) The geometry of the AMC antenna. (**e**) S11 of the AMC antenna. (**f**) Gain of AMC antenna. (**g**) Radiation patterns. (**h**) S11 of the antenna on bending. Reprinted (**a**–**h**) from Ref. [29].

In [29], Mersani et al. proposed a rectangular wearable antenna to discover malignant tumors without actual physical contact. The antenna’s design is divided into two segments. Initially, the monopole antenna (Figure 11a) is fabricated using felt having *ε_r_* equal to 1.22, tanδ equal to 0.016, and thickness h = 2 mm. Electrotextile material Zelt is utilized as the conductive material having a conductivity of 1 × 10^6^ S/m and a thickness of 0.06 mm. As shown in Figure 11b, this antenna’s |S11| has an impedance bandwidth of 4 GHz, and its maximum gain is 2.3 dB (Figure 11c). Secondly, an AMC consisting of square conductive components with annular slots is designed (Figure 11d) to mitigate the effect on the human body. When AMC is introduced to the monopole antenna, the antenna’s BW characteristics improve (Figure 11e). The maximum gain improved from 2.3 to 7.04 dB, as depicted in Figure 11f. In the far field radiation pattern, the antenna is OMD at resonant frequencies with or without AMC, as shown in Figure 11g. The computed SAR is 0.102 W/kg, far lower than the FCC-mandated limit. As illustrated in Figure 11h, when an antenna is subjected to bending, its reflection coefficient magnitudes are slightly less than those of a planar antenna. However, AMC structures generally operate in a narrow band as the final overall design operates between 8.2 and 13 GHz.

## 4. Flexible UWB MIMO Antenna

UWB antennas offers a wide range of applications because of their unique qualities of high data transmission rate, low power consumption, low cost, and excellent reliability [56,57,58,59,60]. Despite these advantages, UWB antennas have drawbacks, such as multipath fading and channel capacity, that affect the system’s overall performance [19,67,68,69]. Recently, the MIMO techniques for UWB systems have gained a great deal of attention because they can fully benefit from the rich diversity provided by copious multipath to improve system performance and overcome the UWB system’s limitations [67,70]. However, the key challenges in MIMO antenna design are maintaining small dimensions, high radiation efficiency, minimal envelope correlation, and good isolation [67,68,69,70,71]. The MIMO antennas require being assessed with the additional metrics referred to as MIMO diversity parameters compared to the traditional antennas. The MIMO diversity parameters are envelope correlation coefficient (ECC), total active reflection coefficient (TARC), channel capacity loss (CCL), diversity gain (DG), mean effective gain (MEG), and the multiplexing efficiency (ME) [67]. The acceptable values of the MIMO diversity parameters for real-time applications are listed in Table 4. Numerous strategies have been proposed to address these obstacles, including placing radiating elements at great distances, resulting in larger antenna dimensions [19,67,68]. Utilizing defective ground structures (DGS), electromagnetic bandgap (EBG), the inclusion of stubs, coupling networks, and neutralizing lines [NL] are different strategies utilized to reduce mutual couplings in UWB MIMO systems [67]. Due to the trade-offs between the enumerated features, achieving such a design would be difficult. Table 5 outlines the performance and cutting-edge designs for flexible UWB MIMO antennas.

**Figure 12:**
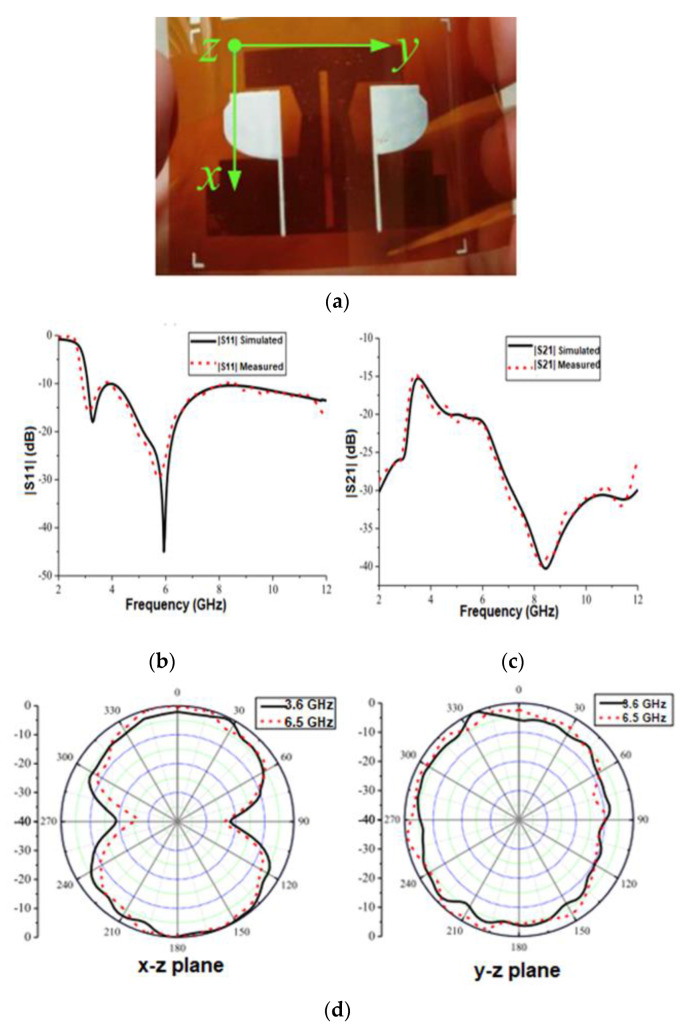

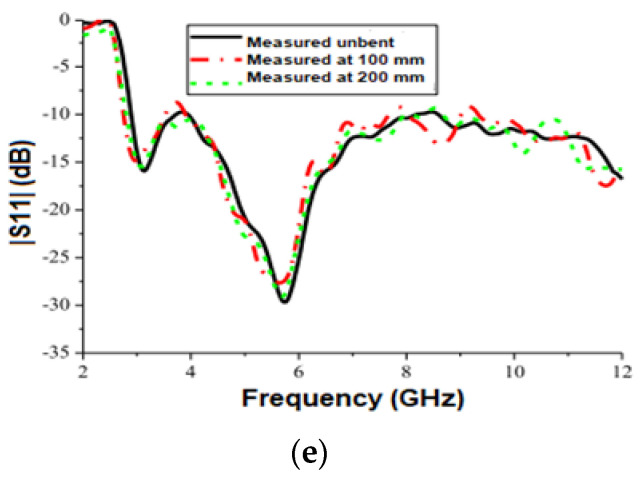
(**a**) The fabricated antenna prototype. (**b**) |S11| curve of the antenna. (**c**) |S21| curves of the antenna. (**d**) Measured radiation pattern. (**e**) Calculated |S11| for a flexible UWB MIMO antenna under bending. Reprinted (**a**–**e**) from Ref. [19].

The literature demonstrates different approaches for enhancing the isolation between the inter-element ports, such as DGS and NL, embedding parasitic elements onto the radiator and ground plane [67]. Li, W. et al. [19] suggested a conformal inkjet-printed dual-element UWB MIMO antenna (Figure 12a). The radiating structure comprises two half-planar monopoles to reduce antenna size. In addition, a redesigned T-shaped stub is added to the ground surface to enhance impedance matching. This antenna’s |S11| shows an impedance bandwidth of 2.9 to 12 GHz (Figure 12b). A slot is etched on this modified T-shaped ground stub to obtain isolation of more than –15 dB (Figure 12c). The antenna has an omnidirectional pattern in the xoz and yoz planes at 3.6 GHz and 6.5 GHz (Figure 12d). The ECC value is observed to be under 0.3, while the CCL obtained is less than 0.4 bit/s/Hz. However, when the bending experiment is performed on foam with varied cylinder radii, S11 deviates slightly (Figure 12e).

Rekha S. et al. [68] presented a two-element UWB MIMO antenna for wearable applications. The substrate for the antenna design with tanδ = 0.02 and *ε_r_* = 2—the highly affordable and extensively utilized denim material—is chosen. The radiator consists of two square patches with horizontal and vertical cuts and a partial ground plane to cover the entire UWB spectrum. The antenna has an impedance BW of 3–11.7 GHz. A redesigned E-shaped stub is positioned between the back surfaces of the radiators to enhance port isolation. The antenna exhibits an OMD pattern in free space and a dipole-like pattern during on-body measurements. SAR analysis is performed at multiple frequencies by varying the distance between the antenna and the human body to confirm the antenna’s safety and usability as a wearable device. At 9 GHz, the SAR factor is less than 1.27 W/kg over 1 g of tissue.

**Figure 13:**
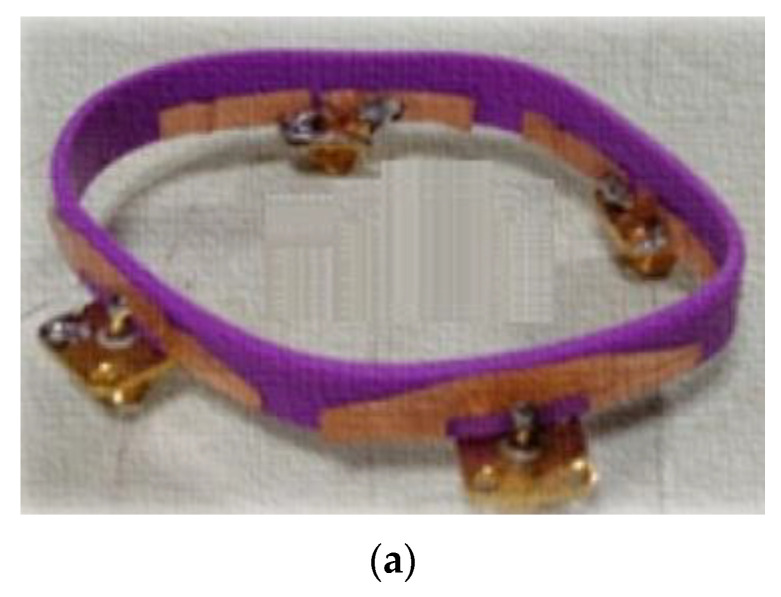

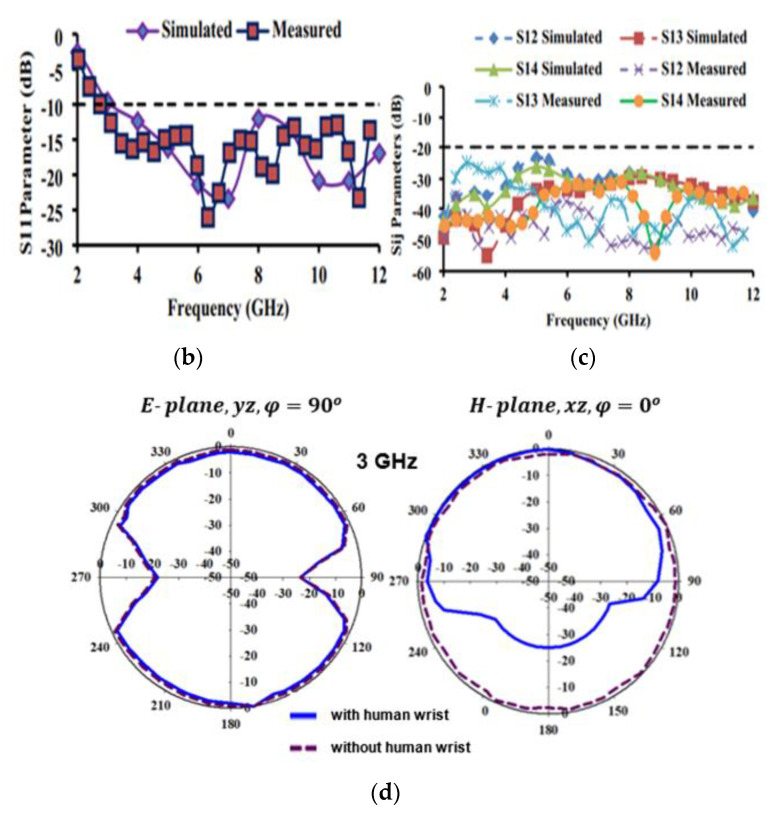
(**a**) The antenna layout. (**b**) S11 curves of the antenna. (**c**) Isolation curve of the antenna. (**d**) Radiation pattern. Reprinted (**a**–**d**) from Ref. [69].

Govindan T. et al. [69] developed a wristband antenna made of silicone rubber that can endure temperatures between −100 and 250 °C. (Figure 13a). UWB bandwidth is achieved using a modified trapezoidal-shaped patch with a partly rectangular ground plane. To further enhance the BW, the slot width close to the feed line is widened, and a rectangular slot is inserted into the ground layer. The space between antenna elements is fixed at 7.5 mm to increase isolation. The impedance bandwidth of 2.75–12 GHz for both simulations and measured (Figure 13b), and isolation more significant than 20 dB, was achieved (Figure 13c). The antenna peak realized gain is 3.41 dBi and the radiation efficiency is 89.3%. The far-field radiation pattern of the antenna is omnidirectional regardless of the position of the user’s wrist (Figure 13d). The SAR of the antenna is studied to assess its radiation exposure to the human body, and it is determined to be below 0.02 W/kg.

Another UWB [70] antenna is fabricated using a denim material with *ε_r_* of 1.6 and a tanδ of 0.02. By using rectangular copper patches and a partial ground layer, the monopole UWB antenna is developed. Two I-shaped stubs are joined in series and positioned on the rear surface to reduce mutual coupling. The antenna has an impedance BW of 1.83 to 8 GHz, minimum element isolation of 22 dB at 2.4 GHz, and peak element isolation of 53 dB at 5.92 GHz. The antenna’s maximum realized gain is 4.4 dB at 6.4 GHz. The antenna has an omnidirectional radiation pattern with low backside radiation. The antenna exhibited stable resonance characteristics and isolation when placed on a human body.

Dey, A. et al. [71] designed a conformal UWB MIMO antenna for wearable applications utilizing a denim substrate with a *ε_r_* = 2 and tanδ = 0.02. A bandwidth ranging from 1.85 to 13.05 GHz is achieved by optimizing the partial ground and dual ring-shaped radiating patch. Two inverted U-shaped stubs are placed on a partial ground layer to reduce mutual coupling. This increases bandwidth to 1.68–13.74 GHz and isolation of 21.7 dB at 8.8 GHz and 51.74 dB at 12.6 GHz. Due to the lossy characteristics of human tissue, the antenna emits directional radiation in the E-plane and H-plane. The computed SAR is significantly below the FCC-mandated limit. However, when the separation between the MIMO antenna and the human tissue model increases, the SAR decreases from high to low values. When the antenna is fixed on the body, the S parameter shows that the antenna provides entire UWB bandwidth and port isolation. In free space, the antenna’s efficiency is 84%. However, the efficiency drops to 73.2% when loaded on the human body.

Govindan T. et al. [72] presented a four-port MIMO UWB antenna design for WBAN applications. The antenna has a hexagonal patch with a partial ground plane that is 4 mm long and is fed by a microstrip line. The bandwidth is improved from 3.08–6.48 GHz to 3.1–12 GHz by inserting a 4 mm stub near the radio frequency transmission line and two cuts in the patch. To reduce mutual coupling, each port faces orthogonally to the others with a 6 mm separation between the antennas. The antenna’s radiation pattern in free space and the human body is omnidirectional. The ECC is less than 0.1, while the CCL is lower than 0.2 bits/s/Hz. The calculated maximum values for efficiency and gain are 98.5% and 3.957 dBi, respectively. Diversity gain is also greater than 9.6 dB in most cases. SAR analysis is carried out so that humans are not exposed to hazardous radiation. The SAR values achieved for 1 g of tissue are 0.513 W/Kg at 4 GHz and 0.316 W/Kg at 8 GHz, respectively.

**Figure 14:**
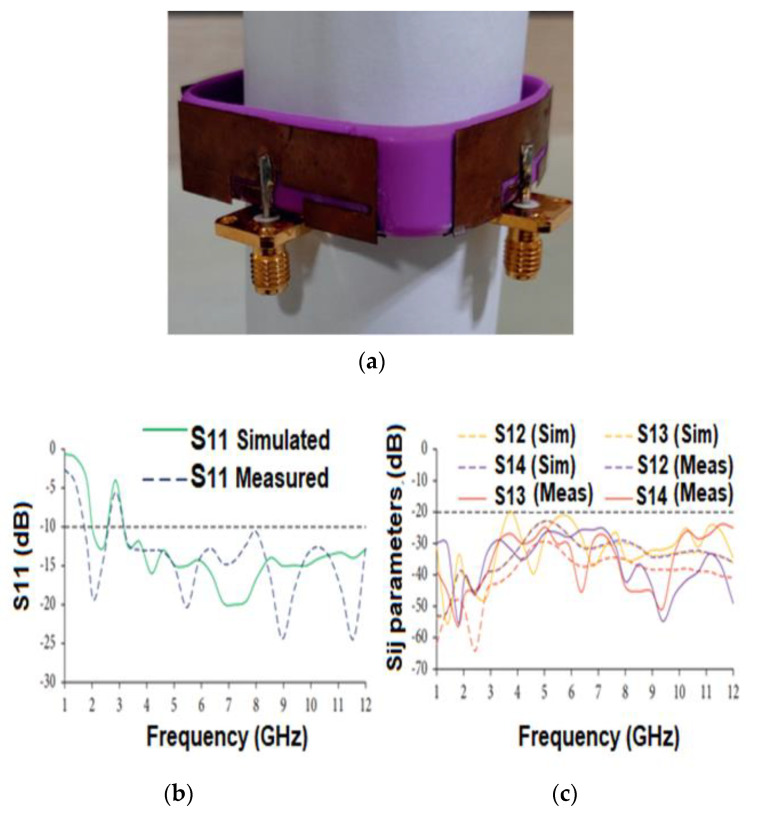

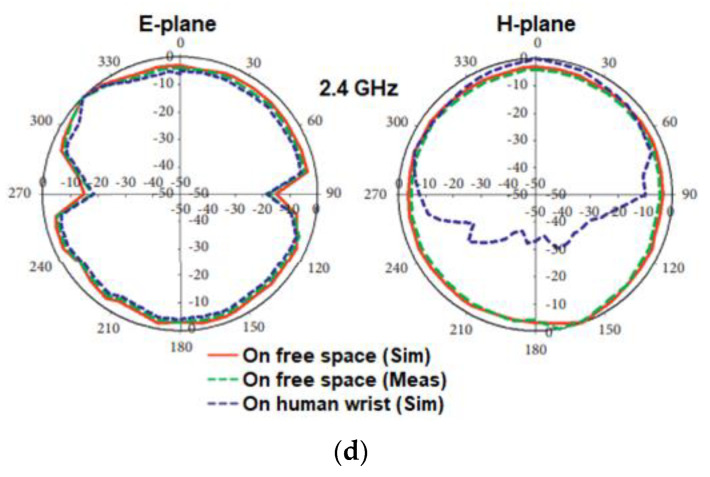
(**a**) The antenna layout. (**b**) S11 curves of the antenna. (**c**) Isolation curve of the antenna. (**d**) Radiation pattern. Reprinted (**a**–**d**) from Ref. [74].

Another UWB antenna [73] is developed using denim material having *ε_r_* = 1.6 and tanδ = 0.02. Two ring-shaped patch elements with a partly etched ground plane are utilized to create the monopole UWB antenna. To reduce mutual coupling, 8 shape stubs are joined in series and positioned at the ground layer. The antenna’s bandwidth is 2.74 to 12.33 GHz, and isolation exceeds 26 dBi. The antenna’s far-field radiation pattern is monopole at 3.8 and 5.8 GHz, but exhibits dipole radiation characteristics at 8.5 GHz. The antenna exhibited stable radiation characteristics and isolation when put on a human body. Govindan T. et al. [74] proposed a rectangular monopole antenna with a DGS, as shown in Figure 14a. To enhance impedance matching, rectangular slots are cut on either side of the radiator, and U-shaped slots are added to the ground layer. To achieve additional resonance at 2.4 GHz, a meander line and a rectangular cut are added to the patch and the ground layer. The antenna is mounted over a silicon rubber substrate with *ε_r_* = 2.9, a tanδ = 0.358, and a bending radius of 16.0825 mm. Simulated and measured impedance bandwidths are found to be 3.1 to 12 GHz (Figure 14b). By keeping elements 0.07 apart, 20 dB of isolation is accomplished (Figure 14c). On the human wrist, an omnidirectional radiation pattern with decreased back radiation is noted, as represented in Figure 14d. The overall efficiency is greater than 95%. Analyses of SAR at various frequencies revealed values substantially below the safety limit.

In [75], the authors designed a quad port single radiator MIMO footwear antenna for wearable applications. The monopole radiating structure comprises a circular ground and radiating patch with a gap G between them. A coaxial port feeds the circular patch and the ground plane. Overlapping circular structures of single elements further developed a MIMO antenna to create a square-shaped radiator, and each element’s ground plane is orthogonal. To increase the bandwidth to 2–14 GHz, the corner of the square antenna is rounded and truncated. Truncating the circular ground near the patch provides 21.5 dB isolation between adjacent ports and improves impedance matching. Both flat and curved antennas are investigated. The antenna has a 2–14 GHz bandwidth and >15 dB flat and >20 dB bent isolation. Cross-polar patterns grow with frequency, while co-polar patterns are BD at 0° and circular at 90°. The SAR is less than 1.6 W/Kg at various resonating frequencies within the UWB range, hence lower than the accepted human safety and health guidelines.

Bisis, A. et al. [76] designed a wearable antenna using a jeans substrate having *ε_r_* = 1.6 and tan(δ) = 0.02. A monopole UWB antenna is developed utilizing a circular patch with a partly etched ground plane. The neutralization line is attached to the patch elements to enhance isolation between the elements. The antenna offers a BW of 3.14 to 9.73 GHz with isolation greater than 32 dB over the entire UWB BW. The antenna’s maximum measured gain is 2.7 dB at 6.4 GHz. The antenna emits directed radiation but produces a minor back lobe in the E plane at 3.7 and 4.2 GHz. The antenna exhibits stable resonance characteristics and isolation when put on a human body.

## 5. Challenges of Designing a Flexible Antenna

The antenna is one of the essential components of the WBAN, which is integrated with smart wearables and clothes to offer reliable wireless connectivity between wearable devices for various applications. One major challenge in antenna design is the need for flexibility due to variations in antenna topology due to the shape of the human body. Selecting suitable conductive and substrate materials that exhibit good electrical and mechanical properties and provide high antenna performance is crucial in designing any flexible antenna. Therefore, selecting the appropriate substrate for a flexible antenna usually involves making trade-offs between thickness, performance, and flexibility. Human–antenna electromagnetic interaction is another critical challenge. In addition, the human body is a sizable inhomogeneous medium with significant biological tissue loss and permittivity. This affects the antenna reflection coefficient, polarization mismatch, radiation pattern distortion, efficiency reduction, and system fidelity factor [6,62]. Defective ground planes or CPW techniques are usually favored because they permit easy BW enhancement. However, these antenna configurations result in back radiation, which inevitably increases SAR within the human body [66]. An SAR analysis with the right anticipated antenna distance from the human body is another primary concern when designing WBAN antennas. In wireless body area networks, the antenna’s radiation pattern and its effect on the measured path gain are also crucial factors. Additionally, the antenna’s surroundings must also be considered. WBAN antenna design must also consider user weight loss/gain, posture, and skin ageing. Additionally, the limitations of dimension, geometry, and surroundings must be considered. The location of an antenna on the body also affects its size and shape, limiting the designer [18]. In addition, skin tissue, muscle, and fat change the characteristics relative to the heating impacts of the electric field and must be considered in the design of WBAN antennas.

## 6. Conclusions

In future, WBAN gadgets will be utilized for daily activities and general well-being in the fields such as healthcare, telemedicine, defense, sports, entertainment, search-and-rescue emergency operations, etc. Furthermore, with the introduction of advanced technologies, such as the IoT and 6G communications, the significance of WBAN is growing and gaining popularity. The next generation of wearable electronics is likely to be miniaturized, inexpensive, lightweight, energy-efficient, low-power, portable, easily accessible, flexible in terms of integration, and equipped with high data rates and superior wireless communication. Due to these critical features, UWB technology is being used as it can support short-distance communication with high-speed, low power consumption, and less interference with other devices. This paper presents a comprehensive overview of flexible UWB antennas for WBAN applications, focusing on the selection of conductive and substrate materials required for their fabrication. It is evident from the literature that the selection of a suitable substrate is crucial to the performance of flexible antennas. It is concluded that the effect of the human body on the antenna exhibit fluctuations in reflection coefficient, gain, bandwidth, and SAR, making the design of a flexible UWB antenna challenging for WBAN application. To enhance channel capacity, system reliability, and transmission speed of data between the components, flexible UWB MIMO antennas are explored. The close placement of the antenna elements results in mutual coupling issues. The decoupling structures can be appropriately designed and utilized for neutralizing the mutual coupling issue in the UWB MIMO antenna system. The design challenges of flexible antennas are also discussed in this article.

## Figures and Tables

**Table 1:** Conductive materials.

Conductive Materials	Conductivity (S/m)	Thickness (mm)	Ref.
**(a) Pure Metal**			
Copper Tape	1 × 10^6^	0.75	[23]
Silver	2.2 × 10^6^	0.007	[22]
Aluminum Tapes	-	0.035	[22]
Copper Sheet	-	0.193	[24]
**(b) Metal Plated Textile**			
Nylon covered with Ni/Cu/Ag	1.02 × 10^5^	0.13	[25]
Nickel–Copper coated fibers	5.4 × 10^4^	0.08	[26]
Meshed Polyester Fibers	2 × 10^5^	0.057	[27]
Copper polyester taffeta	2.5 × 10^5^	0.08	[28]
Zelt	1 × 10^6^	0.06	[29]
ShieldIt	1.18 × 10^5^	0.17	[30]
Nora	-	-	[31]
Flectron	-	-	[32]
Nonwoven conductive fabrics	2.22 × 10^5^	0.15	[33]
**(c) Conductive Polymers**			
Polyaniline (PANI)	4500	0.11	[34]
Polypyrrole	2720	0.116	[35]
PEDOT: PSS	16,000	0.007	[36]
CNT/PANI	-	-	[37]
Pt_C/PANI: CSA	65,600	-	[38]
**(d) Conductive Inks**			
Silver nanoparticle	2.2 × 10^6^	-	[12]
Copper nanoparticle	303 × 10^6^	0.01	[39]
Graphene-based ink	0.25 S/square	0.01	[40]

**Table 3:** Comparison of UWB antennas based on various parameters.

Ref. No.	Size (mm^2^)	Operating Frequency Range (GHz)	Substrate	Gain (dB)	SAR (W/Kg) (Frequency GHz)	Efficiency (%)	Methodology	Merits/Demerits	Demerits
[34] 2018	80×67	7.2–9.2	Kapton ($\varepsilon_{r}$ = 3.48, tanδ = 0.002)	3.1	-	-	Ellipse patch with CPW feed.	Simple structure and highly flexible.	Large physical dimensions. Higher cross-polarization components wen antenna is crumpled.
[59] 2020	32×52	4.1–8.0	Graphene film ($\varepsilon_{r}$ = 3.2)	4.1	-	-	Rectangular patch with two “H” shaped slots.	Super flexible having bending insensitive bandwidth.	The antenna has a lesser impedance bandwidth and resonant frequencies shift at different bending scenarios.
[60] 2019	33×50	1.35–16.4	Polyimide ($\varepsilon_{r}$ = 3.5, tanδ = 0.001)	2.8	-	86%	Elliptical-shaped radiating element, fed by a linearly coplanar waveguide with ladder-shaped ground planes.	Wider impedance bandwidth, contains the entire 3.1–10.6 GHz UWB band and relatively compact.	Low gain and bending effect the far-field radiation patterns of the antenna.
[61] 2020	30.4×48	3.06–13.5 15.9–20.5 20.9–22	Kapton Polyimide ($\varepsilon_{r}$ = 3.5)	1.69	-	59%	Circle- rectangular hybrid shaped antenna.	Wider bandwidth and compact design. The proposed antenna maintains wide bandwidth when *ε_r_* changes from 1 to 4.	Low gain and efficiency
[26] 2020	75×75	2.85–8.6	PDMS ($\varepsilon_{r}$ = 2.77, tanδ = 0.02–0.076)	6.2	-	45%	Angular ring circular patch loaded with two rectangular slots.	Stable radiation pattern across the frequency band.	Complex design, very low efficiency and large dimension.
[62] 2021	106×300	0.34–1.4	Polyimide ($\varepsilon_{r}$ = 3.5, tanδ = 0.0027)	>4	-	60%	Flaring ground with arrow section slots on radiating patch.	The antenna is intended for UHF application and highly flexible due to low thickness of the substrate.	Lower bandwidth and large dimension
[43] 2018	33.1×32.7	3.2–30.0	Photo paper ($\varepsilon_{r}$ = 2.85, tanδ = 0.05)	4.87	-	86.60%	Circular patch with double stepped symmetric ground.	Super wideband and high efficiency.	SAR analysis is not studied.
[24] 2022	67×44	1.5–15	PDMS ($\varepsilon_{r}$ = 2.7, tanδ = 0.0134)	6.76	1.1979 (1.8 GHz) 1.376 (2.4 GHz) 1.0696 (0.6 GHz) 0.6966 (4.2 GHz) 0.4046 (4.8 GHz) 0.5206 (5.2 GHz) 0.3293 (5.8 GHz)	-	A fork-shaped antenna with a circular patch at the center and a crescent-shaped slot below the circle relative to the ground plane.	Better radiation characteristics, gain and bandwidth.	Large physical dimensions.
[28] 2020	40×45	1.198–4.055	Polyester fabric ($\varepsilon_{r}$ = 1.7, tanδ = 0.004)	2.9	0.0014 (10 g) (2 GHz)	56.4% to 70.96%	Interdigit-based radiating patch with triangle slot.	Structure of the design is relatively unique. The antenna retains its performance when it is being bent or working in the proximity of tissue-mimicking phantoms.	An interdigit based radiator contribute to the coupling. Gain and bandwidth are small. The radiation patterns of the bent on-phantom antenna are slightly altered.
[63] 2021	29×37.5	4–8	Soda-lime glass ($\varepsilon_{r}$ = 7.3, tanδ = 0.04)	1.2	-	>63%	Circular monopole antenna with dual substrates and proximity coupling fed.	Compact and transparent antenna.	Complex design and low gain
[64] 2020	55×30	1.77–6.95	Kapton ($\varepsilon_{r}$ = 3.5, tanδ = 0.007)	5.9	-	60%	Two inverted L shaped elements with a matching stub and defected ground structure.	Simple to fabricate and highly flexible.	Large dimension with low efficiency. The radiation patterns are slightly affected on bending.
[65] 2018	33×10	2.632–14.57	Felt ($\varepsilon_{r}$ = 1.44, tanδ = 0.044)	4.84	-	68%	Combination of half elliptical shaped patch with metamaterial unit cell array and Partial ground with slot.	Modified conventional rectangular compact radiator covering the UWB spectrum.	Relatively low gain and efficiency.
[66] 2018	32×52	3.68–10.3	PDMS (*εr* = 2.7, tanδ = 0.02–0.07)	4.53	0.147 (5 GHz) 0.174 (7 GHz) 0.09 (9 GHz)	27%	Two arc-shaped patch with full ground plane	Simple structure with full ground to suppress antenna loading and back radiation.	Large size and very low efficiency.
[6] 2021	33×50	7–28	Denim ($\varepsilon_{r}$ = 1.7)	10.5	0.25 (3.8 GHz) 0.7 (5.8 GHz) 1.29 (7 GHz) 2.04 (28 GHz)	96%	Photonic band gap structures and substrate integrated waveguide.	Full ground, large gain and efficiency.	Complex structure.
[29] 2019	30.4×48	8.2–13	Felt (nylon-based substrate) ($\varepsilon_{r}$ = 1.22, tanδ = 0.016)	7	0.0996 (8 GHz) 0.704 (10 GHz) 0.102 (12 GHz)	-	AMC antenna with square conductive elements and annularly shaped slots.	Stable radiation properties and large gain.	High SAR and on bending resonance frequency shift.

**Table 4:** MIMO diversity parameter [67].

MIMO Diversity Feature	Acceptable Values
ECC	<0.5
DG	∼=10 dB
MEG	<–3 dB
TARC	<−10 dB
CCL	<0.4 bps/Hz
ME	<0 dB

**Table 5:** Performance comparison of UWB MIMO antennas.

Ref Year	Isolation Technique	Number of Ports	Size (mm^2^)	Bandwidth (GHz)	Gain (dBi)	Isolation Level (dB)	SAR (W/Kg) (Frequency GHz)	Diversity Parameters					Shape of Isolation Network
								MEG (dB)	TARC (dB)	Diversity Gain (dB)	ECC	CCL (Bit/sec/Hz)	
[19] 2018	Defected ground plane	2	22 × 31	2.9–12	2.31	<−15	-	-	-	-	0.3	0.4	Slot etched on the modified T—shaped on the ground plane
[68] 2022	Defected ground plane	2	55 × 35	3–12	-	<−19	1.27 (9 GHz)	-	-	<9.975	<0.06	-	E-shaped stub at the ground surface
[69] 2021	Antenna placement and orientation	4	12 × 202	2.75–12	3.41	<−25	0.02	<−1	<−10	>9.5	<0.18	<0.1	Distance of 0.07 λ between the elements
[70] 2022	Defected ground plane	2	40 × 70	1.83–8	4.4	<−22	-	<−2.53	<−10	>9.6	<0.01	<0.2	Two “I” shaped stubs in series on the ground plane
[71] 2022	Defected ground plane	2	50 × 35	1.83–13.82	4.21	<−21	0.784 (8 GHz) 0.893 (11 GHz)	<−3	<−10	>9.9	<0.059	<0.35	Two inverted “U” shaped stubs on ground plane
[72] 2022	Antenna placement and orientation	4	58 × 58	3.1–12	3.957	<−16	0.513 (4 GHz), 0.316 (8 GHz)	-	<−12	>9.6	<0.1	<0.2	Orthogonal alignment with a 6 mm gap between the antennas
[73] 2019	Defected ground plane	2	55 × 35	2.74–12.33	6.9	<−26	-	-	-	>9.9	0.1	0.13	8 shaped stubs placed on ground structure
[74] 2022	Antenna placement and orientation	4	40 × 12	(2.1–2.6), (3.1–12)	3.1	<−20	0.308 (2.4 GHZ), 0.329 (3 GHz), 0.543 (6 GHZ), 0.873 (10 GHz)	-	<−10	>9.4	<0.1	<0.25	Distance of 0.07 λ between the elements
[75] 2022	Defected ground plane	4	92 × 92	2–14	7.2	<−15	0.0058 (3 GHz), 0.0089 (5 GHz), 0.0125 (7 GHz)	-	<−10	>9.6	<0.36	<0.4	Truncating circular ground from both sides near the patch
[76] 2019	Neutralization line	2	30 × 50	3.14–9.73	-	<−32	-	-	<−10	>9.8	<0.1	<0.2	Staircase shape parasitic element connect both the antenna

## Data Availability

Not applicable.
